# Variability in the Alignment of Number and Space Across Languages and Tasks

**DOI:** 10.3389/fpsyg.2018.01724

**Published:** 2018-10-04

**Authors:** Andrea Bender, Annelie Rothe-Wulf, Sieghard Beller

**Affiliations:** ^1^Department of Psychosocial Science, University of Bergen, Bergen, Norway; ^2^SFF Centre for Early Sapiens Behaviour (SapienCE), University of Bergen, Bergen, Norway; ^3^Department of Psychology, Freiburg University, Freiburg, Germany

**Keywords:** number, space, space-number mapping, mental number line, frames of reference, culture, language

## Abstract

While the domains of space and number appear to be linked in human brains and minds, their conceptualization still differs across languages and cultures. For instance, frames of reference for spatial descriptions vary according to task, context, and cultural background, and the features of the mental number line depend on formal education and writing direction. To shed more light on the influence of culture/language and task on such conceptualizations, we conducted a large-scale survey with speakers of five languages that differ in writing systems, preferences for spatial and temporal representations, and/or composition of number words. Here, we report data obtained from tasks on ordered arrangements, including numbers, letters, and written text. Comparing these data across tasks, domains, and languages indicates that, even within a single domain, representations may differ depending on task characteristics, and that the degree of cross-domain alignment varies with domains and culture.

## Introduction

It has long been proposed that humans tend to represent abstract domains such as time or number in terms of more concrete domains such as space (Lakoff and Johnson, 1980). Indeed, evidence for this cross-domain mapping has accumulated over the past 25 years (e.g., Boroditsky, 2000; Fischer and Fias, 2005; Núñez and Cooperrider, 2013). Temporal sequences and events, for instance, appear to be represented along a spatially extending *mental time line* (MTL), as attested to both in linguistic and non-linguistic tasks (overview in Bonato et al., 2012; Bender and Beller, 2014). Likewise, numbers appear to be represented along a spatially extending *mental number line* (MNL), as attested to in tasks using both explicit and implicit measures, such as those concerned with number line estimations (Siegler and Opfer, 2003; Moeller et al., 2009) or with the *spatial–numerical association of response codes* (SNARC) effect (Dehaene et al., 1993; Wood et al., 2008). MTL and MNL have in common that they are assumed to extend in a more or less spatial manner, along one dimension, in one direction, and potentially ad infinitum. An increasing body of evidence related to these constructs seems to corroborate that the domains of space, time, and number are intrinsically linked in human minds, and perhaps even in human brains (Walsh, 2003).

Yet, some observations appear to be at odds with such linear representations, pointing to the possibility that these representations might be neither innate nor universal (e.g., Núñez, 2008, 2011; Bender and Beller, 2014). In particular, three sets of findings are inconsistent with a simply painted picture of cross-domain congruency: (1) the remarkable degree of variability in representations, both within and across domains; (2) the deep impact of cultural practices on the shape of these representations; and (3) the dependency of such representations on task specifics and context.

### Variability in representations

Time is the prototypical example of how variable the spatialization of abstract concepts may be (for overviews, see Galton, 2011; Bender and Beller, 2014). Besides the *linear* representation, which has invited the image of a mental time line, time can also be represented as cyclically recurring (Le Guen and Pool Balam, 2012) or as radially extending from (or pointing toward) one's own present (Bennardo, 2009; Bender et al., 2010). The latter concept in particular, with its half-axes radiating out from the conceptual (deictic) center, factually precludes the existence of a single time line. And it is claimed that some groups like the Yucatec Maya or Amondawa do not represent time in terms of space at all (Sinha et al., 2011; Le Guen and Pool Balam, 2012).

But even those representations that are compatible, in principle, with a linear spatial construct may still vary regarding the number of different time lines a person can hold (e.g., Miles et al., 2011); regarding the axis (i.e., lateral, sagittal, or vertical) along which the lines unfold and the direction in which they point (e.g., Fuhrman et al., 2011; Bergen and Chan Lau, 2012); and regarding whether the lines are anchored in the speaker's subjective present or in objective features of, say, the landscape (e.g., Boroditsky and Gaby, 2010; Núñez et al., 2012a). Part of this variability is due to the fact that, also for spatial representations, we do not just have one available option, and our preferences depend on a bunch of partly unrelated factors, including the perspective focused on and the affordances and constraints inherent in the tasks used.

Whether a similar degree of variability may also be found for the MNL has not yet been investigated in a systematic manner, but possible sources and types of variation have been discussed (e.g., Galton, 1883; Ernest, 1986; Bender and Beller, 2011; Núñez, 2011; Winter et al., 2015), and some characteristics of the MNL are known to vary due to cultural influences (see next section). Yet, with the remarkable degree of variability even *within* one domain, it is almost obvious that there cannot be a simple congruence of the spatially grounded, mental representations of time (MTL) and number (MNL) *across* domains either.

### Cultural impact

A great deal of the variability reported in the previous section can be accounted for by cultural influences, including linguistic metaphors, culture-specific concepts, and culturally embedded practices. For instance, not only the choice of a specific conceptualization of time (i.e., as linear, cyclical, or radial), but also the dimension and direction of linear representations are affected by cultural beliefs and epistemological frameworks, implying, for instance, how the future relates to the present, or whether the future is located in front of or behind the speaker (e.g., León-Portilla, 1990; Núñez and Sweetser, 2006). If time is represented along a linear axis, its direction appears to be additionally influenced by linguistic metaphors, as reflected in expressions such as “looking forward to the future” (inviting a sagittal line pointing from back to front) or “a custom handed down to us from our ancestors” (inviting a vertical line pointing downwards). Moreover, cultural practices such as those underlying the preferential reading and writing direction appear to be correlated with the direction of the time line (e.g., Tversky et al., 1991; Bergen and Chan Lau, 2012).

A similar influence of the reading and writing direction has also been observed for the mental number line, which extends from left to right for speakers of English, but from right to left for speakers of Arabic and Hebrew (Dehaene et al., 1993; Zebian, 2005; Shaki et al., 2009; for an additional or alternative influence of finger counting, see also Fischer and Brugger, 2011; Bender and Beller, 2012). The second feature of the MNL which is subject to cultural influences is its scale: initially logarithmic, it seems to shift toward linearity with the extent of formal mathematical education (Dehaene et al., 2008), even though some interpret the available data as a composition of two distinct number lines rather than the transformation of one into another (e.g., Moeller et al., 2009).

Finally, both for time and for number, representations may not be spatialized at all (overview in Bender and Beller, 2014), at least not along a spatially extended line. As convincingly argued by Núñez (2008, 2011), the number line is actually a highly sophisticated and culturally mediated concept that took centuries to develop in a particular cultural and historic context, strongly linked to cultural practices of measuring and to instruments such as rulers. Once in place, these practices give rise to SNARC-like effects, not only for quantity representations, but for all kinds of sorting tasks, also for non-numerical categories (Núñez et al., 2011). In untrained participants, at least some response patterns are more accurately accounted for by nonlinear representations (Núñez et al., 2012b), and the extent to which they are spatial to begin with partly depends on the task used (see next section). Clearly, there is a dire need for more research into the exact nature of number representations not affected by Western schooling (Beller et al., 2018).

### Dependency on tasks

A third complication in the picture of cross-domain congruency emerges from the observation that response patterns may depend on task specifics and context.

Again, let us begin with the domain of time, for which this has been analyzed in detail. The toolkit of tasks used to investigate spatial representations of time includes different paradigms: language elicitation, observation of co-speech gestures and postural sway, mapping tasks, and reaction time paradigms based on congruency priming. Notably, the observed time line was found to differ profoundly in terms of the axis along which it unfolds, depending on the paradigm used to investigate it. English speakers, for instance, exhibit a sagittal time line in linguistic tasks and when measuring postural sway, but rely almost exclusively on the lateral axis in co-speech gestures and for tasks requiring spatial layouts (for an overview, see Bender and Beller, 2014). Even within a single paradigm, specifically the tasks based on congruency priming, a variety of axes can be activated depending on task-specific characteristics (Torralbo et al., 2006).

Response patterns may also depend on whether the task explicates the issue of mapping or leaves it implicit. Normally, space-time mappings appear to be highly automatized. Co-speech gestures, for instance, are produced spontaneously without people necessarily being aware of them. In such cases, English speakers strongly prefer to recruit the lateral axis, with leftward gestures for earlier times and rightward gestures for later times. If these same people are asked to *deliberately* produce gestures referring to past and future events, however, they do so much more often along the sagittal axis, familiar to them from linguistic metaphors (Casasanto and Jasmin, 2012).

Crucially, apart from the purely linguistic tasks, most of the tasks typically used in this field contain a spatial component: Co-speech gestures and postural sway inevitably unfold in space, and this is also true for abstract pointing and the arrangement of tokens required in mapping tasks and for the predefined congruency priming in reaction time paradigms. It is thus not surprising that these tasks uncover *spatialized* representations of time. The same arguably holds for the domain of number, where both SNARC effect studies and number line estimation tasks *a priori* impute the spatial representation they try to measure (for related arguments, see also Núñez, 2011; Shaki and Fischer, 2018).

In other words: Spatial representations of number—as of time—may be more diverse than we tend to assume; but in order to explore this realm of possibilities, we require tasks that allow participants to recruit other dimensions beyond the well-known number line; we need to pay attention to perspective and frames of reference; and we need to take the diverse sources of linguistic and cultural variability more seriously. The study reported here, while exploratory in nature, is intended as a first step in this direction. It is based on a paper-and-pencil survey conducted among speakers of five languages: English, Norwegian, German, Chinese, and Japanese.

## The study

Given that both time and number appear to be represented in terms of space, the study reported here aims at exploring the extent to which spatial representations of number may be subject to the same degree of variability, cultural impact, and dependency on task specifics and context as are spatial representations of time. To this end, the study focuses on the extent to which spatial representations of symbolic number depend (i) on a particular perspective or frame of reference, (ii) on the linguistic and cultural background of participants, and (iii) on a specific task.

Regarding (i), spatial representations and inferences change fundamentally depending on which perspective is taken, that is, whether a superordinate field, a given reference point, or a subjective viewpoint is taken as the underlying frame of reference (Levinson, 2003; Majid et al., 2004; Haun et al., 2011). Representations also change depending on whether objects are at rest (static) or moving (dynamic), with assignments of front and forward sometimes flipping between tasks that require a token either to be picked or moved (Bender et al., 2012). While similar dependencies have been observed for temporal representations, little is known about whether they may also be in place for number representations. We therefore collected data for fixed (static) relations vs. changing (dynamic) relations between specified numbers and number sequences, and data on whether a spatial orientation can be assigned to number sequences and the number line itself.

Regarding (ii), as preferences for a specific frame of reference in the domain of space do vary substantially across languages and cultures (Senft, 1997; Majid et al., 2004; Beller et al., 2015; Beller and Bender, 2017), a corresponding variability in number representations should be observed if these are really grounded in spatial representations. We therefore collected data from speakers of several languages that differ not only in writing systems, traditional writing direction, patterns of finger counting, and/or composition of number words (as detailed below), but also in their preferences for spatial frames of reference.

And regarding (iii), while spatial representations of number yielded with number line estimation tasks and in SNARC studies are necessarily confounded with the spatial layout of the tasks themselves, linguistic tasks offer more leeway for participants to provide responses that need not be compatible with a spatial representation. We therefore collected linguistic data in a questionnaire that explicitly asks participants, in different ways, about the orientation of numerical representations.

### Selection of languages

The survey was conducted with speakers of five languages: English, Norwegian, German, Chinese, and Japanese. English, Norwegian, and German belong to the Germanic branch of the Indo-European language family. While the two East Asian languages in our sample have no “genetic” relationship, Japanese has been influenced by Chinese in several ways, including with regard to the writing system, parts of the vocabulary, and the number system. The languages were chosen because they differ on several potentially relevant dimensions, including traditional writing direction, preferences for spatial and temporal referencing, and properties of the counting systems.

#### Writing systems in the selection of languages

**Figure 1:**
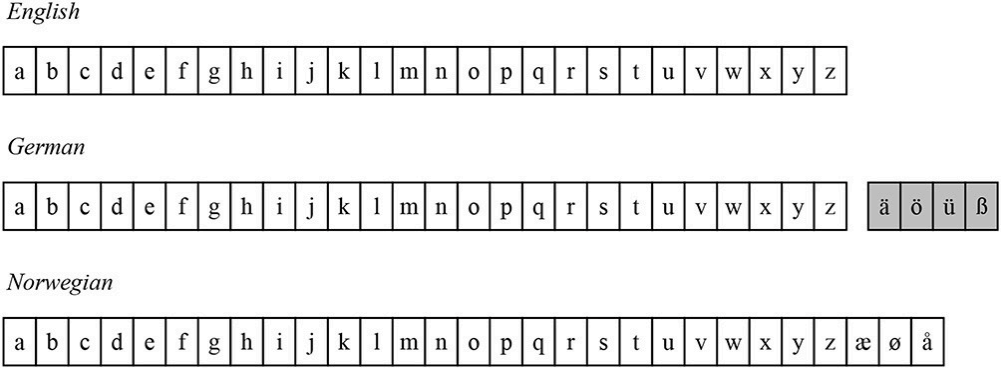
The English, German, and Norwegian alphabet (enumeration of the German alphabet typically includes the basic letters only; the *umlauts ä*, ö, and ü, and the *eszett, ß*, are important for writing, but lack canonical position in the alphabetical order, hence underlaid in gray here).

The writing systems of the Germanic languages are based on Latin, with a few additional letters in the case of Norwegian and German. The alphabet begins with “a” in all three languages, and ends in “z” in both English and German, and in “å” in Norwegian (Figure 1). All three are written from left to right, with lines ordered from top to bottom.

**Figure 2:**
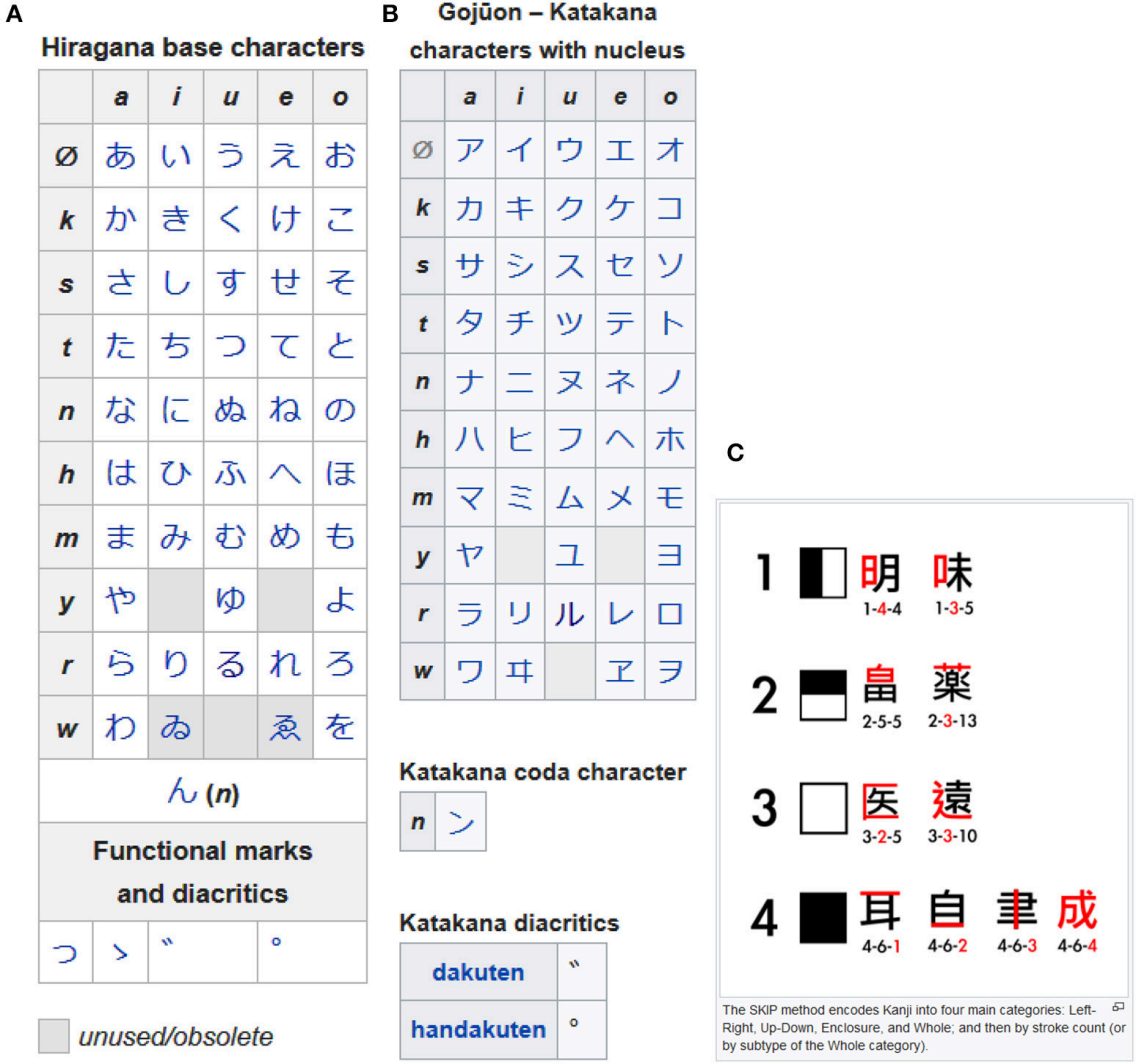
Japanese writing systems: the two syllabaries *hiragana* **(A)** and *katakana* **(B)** in the *gojuon* ordering, and some of the *kanji* logograms **(C)** illustrating one of the methods for sorting them. Sources: **(A)** https://en.wikipedia.org/wiki/Hiragana, **(B)** https://en.wikipedia.org/wiki/Katakana, **(C)** https://en.wikipedia.org/wiki/Kodansha_Kanji_Learner%27s_Dictionary (all retrieved on Sep 7, 2018); the *Illustrations of the SKIP method as described in en: Kodansha Kanji Learner's Dictionary* was created by Babbage (2011) and is licensed under the “Creative Commons Attribution-Share Alike 3.0 Unported license” (https://commons.wikimedia.org/wiki/File:SKIP_Kanji_method_examples.svg).

The standardized form of spoken Chinese is written with logograms (i.e., Chinese characters) in one of two versions: the Simplified Chinese character system prevailing in the People's Republic of China, and the traditional system used outside mainland China. The written standard of Japanese uses mainly two types of writing systems: a set of logograms based on Chinese characters (*kanji*) and two syllabic scripts (*kana*). For the two syllabaries, *hiragana* and *katakana*, the modern and prevalent ordering system *gojuon* is based on 2-dimensional tabulation, beginning with vowel “a” in the upper left corner and ending on “wo” in the bottom right corner (Figures 2A,B). By contrast, *kanji*, with its thousands of symbols, resists conventional ordering and is therefore sorted according to the composition principles on which the characters are based (for an example, see Figure 2C). Traditionally, Chinese and Japanese were written from top to bottom, but writing from left to right is becoming increasingly frequent.

#### Representations of space and time in the selection of languages

For describing spatial relations, speakers of all five languages make use of all known basic frames of reference (FoR), but differ with regard to the variant of the relative FoR. These variants differ in how the coordinate system that informs the viewpoint of an observer is transferred to the reference point for localizing an object in relation to the reference point. The object “in front of” the reference point would be the nearer object in the reflection variant, but the further-away object in the translation variant (Levinson, 2003). Germans strongly prefer the reflection type, while the others use both the reflection and translation type, but in distinct proportions (Beller et al., 2015; Beller and Bender, 2017; and see Bender et al., 2012).

With regard to time, speakers of most of these languages appear to recruit at least two distinct axes, and one of these in both directions, depending on task and context (data on English, German, and Chinese are reviewed in Bender and Beller, 2014; for data on Norwegian, see Bender et al., 2017; relevant data on Japanese are still lacking). All recruit the *sagittal* axis with a preference for back-to-front when representing past versus future events, yet with a preference (among German and Chinese speakers) or ambivalence (among English and Norwegian speakers) for the reversed front-to-back when events are moved forward. The *lateral* axis left-to-right is additionally or exclusively used in tasks that recruit space as the medium for representing time, such as in sign language or on paper. Finally, Chinese speakers also recruit the *vertical* axis top-to-bottom.

#### Representations of number in the selection of languages

The number system in each of the five languages is largely decimal, both for number words and for numerical notations, but composition in the Germanic languages is substantially less regular and transparent than in the Eastern Asian languages (cf., Miura, 1987; Calude and Verkerk, 2016). Notations are based almost exclusively on Arabic digits for speakers of the Germanic languages, but to some extent also for speakers of Chinese and Japanese, alongside the more traditional Chinese characters. Studies on the mental number line indicate an alignment of the number line primarily with reading and writing direction, in that speakers of English and German exhibit left-to-right orientation (overview in Göbel et al., 2011) and Chinese speakers left-to-right or top-to-bottom orientation, depending on whether numbers are presented as Arabic digits or as Chinese characters (Hung et al., 2008). Japanese speakers, by contrast, were found to respond with left-to-right and bottom-to-top (Ito and Hatta, 2004). Patterns of finger counting which are additionally or alternatively assumed to affect the mental number line (Fischer and Brugger, 2011) are largely similar for speakers of English, Norwegian, and German, but more different for speakers of Japanese, and even more different for speakers of Chinese (overview in Bender and Beller, 2012).

#### Language-specific possibilities for the spatialization of number

It may thus be expected that, if number representation follows the preferences for spatial representations, gradual differences between the five languages should be observed, in line with the difference in the degree to which speakers of these languages prefer the reflection versus the translation variant of the relative frame of reference. If number representation follows the preferences for temporal representations, German and Chinese should pattern alike, and should be distinct from English and Norwegian. Finally, if cultural and linguistic factors such as the direction of writing and reading or the transparency of number words play a crucial role, then English, Norwegian, and German should pattern alike, and should be distinct from Chinese and Japanese (which may also differ from one another due to different finger counting patterns).

### Methods

#### Samples

A total of 475 individuals participated in this study. Seven participants were excluded due to being non-native speakers in their respective sample.

The English-speaking sample of 62 individuals was recruited at the University of Nottingham, Great Britain. Most participants were students; 33 (53.2%) were female; and their mean age was *M* = 19.8 years (*SD* = 2.6, range 18–36).

The Norwegian-speaking sample of 78 individuals was recruited at the University of Bergen, Norway. Most participants were students; 59 (75.6%) were female; and their mean age was *M* = 25.3 years (*SD* = 7.6, range 19–62; five did not indicate their age).

The German-speaking sample of 116 individuals was recruited at the University of Freiburg, Germany. Most participants were students; 74 (63.8%) were female; and their mean age was *M* = 23.0 years (*SD* = 4.9, range 18–47; two did not indicate their age).

The Chinese-speaking sample of 89 individuals was recruited from the Chinese community in Freiburg, Germany, and from short-term language courses for foreign students at the University of Freiburg. Most participants were students; 62 (69.7%) were female; and their mean age was *M* = 25.5 years (*SD* = 3.4, range 18–38; four did not indicate their age).

The Japanese-speaking sample of 123 individuals, finally, was recruited at Nagoya University, Japan. All participants were students; 41 (33.6%) were female (one did not indicate his or her gender); and their mean age was *M* = 19.5 years (*SD* = 1.8, range 18–34; two not did not indicate their age).

#### Materials

The tasks described in the following were part of a larger paper-and-pencil survey that also included temporal and purely spatial items. Here, we focus only on those items that contain numbers or other ordered sequences such as letters or text segments, which are relevant for the questions under scrutiny in this paper. Letters in the tasks on letters (α1–α4) were based on the Latin alphabet for all but Japanese speakers, for whom the *hiragana* in the *gojuon* ordering was used instead. In the following, we use the British English version for illustration; for translations into Norwegian, German, Mandarin Chinese, and Japanese, see section 1 of the Supplementary Materials. Translations were conducted by bilingual speakers and subsequently back-translated.

(1) The *Moving Task (Mov)* consisted of four items, with an entity to be moved forward or backward (Norwegian: *fram* or *bakover*; German: *nach vorne* or *nach hinten*; Chinese: 往前 or 往后; Japanese: 前に or 後ろに) in the given context. Two items referred to numerical entities:

(Mov_n1) The 7th signpost was moved {forward/backward} by two positions. Which number does the signpost now have?
(Mov_n2) Jenny wanted to marry on the 15th of August, but the date had to be moved {forward/backward} by 7 days. On what date does the wedding now take place?

The other two items referred to other ordered entities:

(Mov_α1) If, in the English alphabet, the letter “E” were moved {forward/backward} by one position, between which two letters would it end up?
(Mov_s1) If, in this sentence, the word “apple” were moved {forward/backward} by three positions, between which two words would it end up?

The items were implemented in four arrangements, crossing, between subjects, two *phrasings* with two *orders of items*. Regarding the phrasings, two items requested a “forward” movement (e.g., *n2* and α1), the other two items a “backward” movement (e.g., *n1* and *s1*), and vice versa^1^. One item order was *n2, n1, s1*, and α1, the other was the exact reversal.

(2) The *Order Task (Ord)* consisted of five items that asked for the order of entities, that is, whether a target entity is in front of or behind (Norwegian: *foran* or *bak*; German: *vor* or *hinter*; Chinese: 前面 or 后面; Japanese: 前 or 後) a reference entity. Two items used a forced-choice format, three used an open format. Two items referred to numerical entities:

(Ord_n3) Number 25 is two positions …
  □ in front of
  □ behind
  … number 23.
(Ord_n4) Which number is 5 positions {in front of/behind} 9?

The other three items referred to other ordered entities:

(Ord_α2) In the alphabet, the letter M is …
  □ in front of
  □ behind
  … the letter P.
(Ord_α3) Which letter is directly {in front of/behind} G in the alphabet?
(Ord_s2) In this sentence, which word is two positions {in front of/behind} the underlined word “two”?

The items were implemented in four arrangements, crossing, between subjects, two *phrasings* with two *orders of items*. The phrasing concerned the order of response options for the forced-choice items (“in front of” first vs. “behind” first) and the preposition used for the open items (“in front of” for the items *n4* and *s2*, and “behind” for the item α*3*, or vice versa). One item order was determined randomly, the second order was the exact reversal.

(3) The *Front Assignment Task (Ass)* consisted of four items that directly asked whether or not an ordered sequence of entities has a front or back (Norwegian: *forside* or *bakside*; German: *Vorne* or *Hinten*; Chinese: 前面 or 后面; Japanese: 前方 or 後方), and if so, in which direction it is pointing. All items followed the same schema and had four response options, exemplified here for the item on the number list:

(Ass_n5) {*Front/Back*} of an ordered number list …
  □ is at the smallest number.
  □ is at the largest number.
  □ Something like this does not exist.
  □ Something else, namely _______.

As the last two response options were the same for all items, we explicate only the item-specific options for the three remaining items on other ordered sequences:

(Ass_α4) {*Front/Back*} of the English alphabet …
  □ is at the letter “a”.
  □ is at the letter “z”.
(Ass_w) {*Front/Back*} of the word “holiday” …
  □ is at the letter “h”.
  □ is at the letter “y”.
(Ass_q) {*Front/Back*} of a questionnaire …
  □ is at the instruction part.
  □ is at the thanking part.

The items were implemented in four arrangements, crossing, between subjects, two *phrasings* (asking for all items either for “*Front* of X …” or “*Back* of X …”) with two *orders of items* (one random order and the exact reversal).

#### Design and procedure

Four versions of questionnaires were constructed. The various types of tasks were presented within subjects in a fixed order (i.e., the Moving Task followed by the Order Task followed by the Front Assignment Task) in line with the increasingly explicit nature of the task (asking for the “front” of an ordered number list highlights the topic of interest more strongly than asking for the date to which an event is moved). The four item arrangements of each task were randomly assigned to one of the four versions of questionnaires, and varied between subjects, as indicated in the Materials section. Participants were instructed to work on all tasks in the given order.

### Results

After some preliminaries describing data coding and the procedure for analyzing the single items, we present three types of analyses, separately for numerical and other (i.e., alphabetical and textual) items: item-level analyses, an analysis of participants' individual consistency across items, and an analysis that helps to decide to what extent the observed variation is task-specific or item-specific.

#### Preliminaries

Our tasks required participants to indicate a moving direction (Moving Task), a succession (Order Task), or an orientation (Front Assignment Task), depending on a specific phrasing (i.e., forward/backward, in front of/behind, and front/back, as described in the Materials section). To enable a comparison of the responses across the phrasings, we re-coded the responses as to whether they indicated that front of the moving direction, of the reference entity, and of the figure's orientation, respectively, points (i) toward the smallest or largest number of a number sequence for the numerical items, (ii) toward the beginning or the end of the alphabet/*hiragana* for the alphabetical items, and (iii) toward the beginning or the end of a written segment for the textual items. For answers that did not allow an unambiguous re-coding of front, a missing value was assigned.

We began the data analysis by testing the responses, for each item separately (*item-level analyses*), for differences between languages, and for possible effects of the phrasings and item orders. To this end, we ran a log-linear analysis (Kennedy, 1992) on the re-coded responses with three independent variables: *language* (five versions), *phrasing* (two versions), and *order* of items (two versions). Main effects and interactions were tested for significance by comparing two log-linear models that differ in one candidate factor only.

The analysis started with the full model including the main effects and interactions of all factors. Then, we simplified the model stepwise by excluding one candidate factor at a time (as the basis for the next comparison) in the following order: (1) *language* × *phrasing* × *order*, (2) *phrasing* × *order*, (3) *language* × *order*, (4) *order*, (5) *language* × *phrasing*, (6) *phrasing*, (7) *language*. We began with those candidate factors that include the order of items, as we did not expect to find effects of this control variable, and then inspected effects of phrasing and language. Fit values of the computed log-linear models and significance values of the various main effects and interactions are reported in section 2 of the Supplementary Materials for each item.

#### Front assignment on numerical items

In the following, we first describe the results of the item-level analyses. Then, we determine participants' individual consistency in assigning front, and inspect possible sources of the observed variation.

*(1) Item-level analyses*. The log-linear analyses indicated a strong main effect *language* for each of the five numerical items, and a modulating effect of how the items were phrased for four items. Participants' front assignments depending on language and phrasing are reported in Table 1.

**Table 1:** Percentage of (*N*) participants assigning Front to the smallest number of a sequence for the five numerical items (n1 to n5), depending on language and phrasing.

		**English**		**Norwegian**		**German**		**Chinese**		**Japanese**	
		**Phrasing**		**Phrasing**		**Phrasing**		**Phrasing**		**Phrasing**	
		**Forward**	**Backward**	**Forward**	**Backward**	**Forward**	**Backward**	**Forward**	**Backward**	**Forward**	**Backward**
**MOVING TASK**											
Mov_n1	Front=smallest^a^	53.3 (30)	51.6 (31)	47.4 (38)	37.5 (40)	86.2 (58)	87.3 (55)	90.9 (44)	95.5 (44)	95.1 (61)	75.4 (61)
Mov_n2	Front=smallest^a^	67.7 (31)	54.8 (31)	40.0 (40)	42.1 (38)	89.7 (58)	98.3 (58)	100 (44)	97.7 (44)	98.3 (121)	– (–)
		**Phrasing**		**Phrasing**		**Phrasing**		**Phrasing**		**Phrasing**	
		**In front of**	**Behind**	**In front of**	**Behind**	**In front of**	**Behind**	**In front of**	**Behind**	**In front of**	**Behind**
**ORDER TASK**											
Ord_n3	Front=smallest^a^	71.0 (31)	41.4 (29)	70.0 (40)	86.8 (38)	96.6 (58)	91.4 (58)	100 (44)	97.8 (45)	83.6 (61)	79.0 (62)
Ord_n4	Front=smallest^a^	80.0 (30)	56.3 (32)	87.5 (40)	59.5 (37)	100 (58)	86.2 (58)	100 (45)	100 (44)	100 (61)	78.7 (61)
		**Phrasing**		**Phrasing**		**Phrasing**		**Phrasing**		**Phrasing**	
		**F****ront**	**B****ack**	**F****ront**	**B****ack**	**F****ront**	**B****ack**	**F****ront**	**B****ack**	**F****ront**	**B****ack**
**F****ront** **ASSIGNMENT TASK**											
Ass_n5	Front=smallest	90.0 (30)	31.3 (32)	61.0 (41)	19.4 (36)	89.7 (58)	77.6 (58)	84.1 (44)	77.3 (44)	93.5 (62)	90.2 (61)
	Front=largest	0.0	15.0	0.0	5.6	3.4	12.1	2.3	0.0	1.6	0.0
	Does not exist	10.0	43.7	34.1	63.9	3.4	8.6	6.8	15.9	4.8	8.2
	Other	0.0	0.0	4.9	11.1	3.4	1.7	6.8	6.8	0.0	1.6

a *Percentage Front=largest is 100 – percentage Front=smallest*.

For the item *Mov_n1*, the main effect *language* (*G*^2^[4] = 88.941; *p* < 0.001) was the only significant effect. Assignment of front to the smallest number was frequent among speakers of German (86.7%), Chinese (93.2%), and Japanese (85.2%), and less frequent among speakers of English (52.5%) and Norwegian (42.3%).

For the item *Mov_n2*, the analysis revealed two significant effects: a main effect *language* (*G*^2^[4] = 154.410; *p* < 0.001) and a small but significant three-way interaction *language* × *phrasing* × *order* (*G*^2^[3] = 9.969; *p* = 0.019). Again, assignment of front to the smallest number was frequent among speakers of German (94.0%), Chinese (98.9%), and Japanese (98.3%), and less frequent among speakers of English (61.3%) and Norwegian (41.0%). The interaction indicated minor moderating effects of the phrasing and item order.

For the item *Ord_n3*, the analysis revealed two significant effects: a main effect *language* (*G*^2^[4] = 61.638; *p* < 0.001) and a small but significant interaction *language* × *phrasing* (*G*^2^[4] = 10.429; *p* = 0.034). Assignment of front to the smallest number was frequent among speakers of German (94.0%), Chinese (98.9%), Japanese (81.3%), and, this time, also Norwegian (78.2%), and less frequent among speakers of English (56.7%). The interaction reflected differences between the two phrasings, mainly for English and Norwegian. For English, assignment of front to the smallest number was more frequent when the item asked whether a number is “in front of” another number (71.0%) than when it asked whether a number is “behind” another number (41.4%). The pattern was reversed for Norwegian: Assignment of front to the smallest number was less frequent when the item was phrased with “in front of” (70.0%) than when it was phrased with “behind” (86.8%). For the other languages, the difference between the two phrasings was only marginal (≤5.2%).

For the item *Ord_n4*, the analysis again revealed two significant main effects: *language* (*G*^2^[4] = 54.526; *p* < 0.001) and *phrasing* (*G*^2^[1] = 34.609; *p* < 0.001). This time, front was preferably assigned to the smallest number in all languages: highly frequent among speakers of German (93.1%), Chinese (100%), and Japanese (89.3%), and less so, but still frequent, among speakers of English (67.7%) and Norwegian (74.0%). Overall, this preference varied with the phrasing of the item: It was stronger when the item asked whether a number is “in front of” another number (95.3%) than when it asked whether a number is “behind” another number (78.4%).

Finally, for the item *Ass_n5*, the analysis revealed three significant effects: two main effects, *language* (*G*^2^[12] = 109.000; *p* < 0.001) and *phrasing* (*G*^2^[3] = 32.832; *p* < 0.001), and an interaction *language* × *phrasing* (*G*^2^[12] = 21.732; *p* = 0.041). Again, assignment of front to the smallest number was frequent among speakers of German (83.6%), Chinese (80.7%), and Japanese (91.9%), and less frequent among speakers of English (59.7%) and Norwegian (41.6%). Overall, this preference was stronger when the item asked participants to indicate the “front” of an ordered number list (smallest: 84.7%; largest: 1.7%; does not exist: 10.6%; other: 3.0%) than when it asked them to indicate the “back” of such a list (smallest: 65.4%; largest: 7.4%; does not exist: 23.4%; other: 3.9%). For a substantial proportion of participants, an ordered number list apparently lacks a front or back. This response was particularly frequent among the English- and Norwegian-speaking participants (27.4 and 48.1%, respectively), as indicated by the significant interaction.

On the whole, the data of the numerical items revealed a quite uniform assignment of front to the smallest number for German, Chinese, and Japanese, and more mixed assignments of front for English and Norwegian. As expected, the control variable *item order* had little influence. The different phrasings played a role in four of the five items, suggesting that the assignment of front to the smallest number was more pronounced when an item asked whether something is “in front of” or is the “front” of a reference entity, but the pattern was not completely homogeneous. Regarding the three types of tasks, the results were fairly homogeneous in all samples except for the Norwegian one; there, the modal response switched from an assignment of front to the largest number in the Moving Task to an assignment of front to the smallest number in the Order Task, and to “Something like that does not exist” in the front Assignment Task.

So far, we have inspected each item separately. In the following, we determine the extent of variation across items by looking at participants' individual consistency, and we determine possible sources of the observed variation by looking at participants' individual response patterns.

*(2) Individual consistency*. In order to obtain an overall measure that reflects the extent to which a participant's responses vary across items, we counted how often front was assigned to the smallest number and how often it was assigned to the largest number, respectively, across the *N* numerical items that a participant had solved^2^. For example, if front was assigned to the smallest number on five out of the *N* = 5 items, consistency would be 100% for “front=smallest”; if front was assigned to the smallest number on three items, to the largest number on one item, and was claimed to be “non-existent” on the final item (Ass_n5), consistency would be 60% for “front=smallest” and 20% for “front=largest”; and if front was assigned to the smallest number on three items and to the largest number on one item out of *N* = 4 items (one missing response), consistency would be 75% for “front=smallest” and 25% for “front=largest.” We then used the *maximum* of the two counts as an estimate of a participant's consistency across the whole set of items (i.e., 100, 60, and 75% respectively in the examples).

Across the five samples, front was assigned to either the smallest or the largest number with a mean consistency of 85.3%. An analysis of variance indicated significant differences between the languages; *F*_(4, 463)_ = 55.212; *p* < 0.001; η^2^ = 0.323. Consistency across the five numerical items was high for the speakers of German (91.7%), Chinese (94.5%), and Japanese (89.4%), and was lower for the speakers of English (71.7%) and Norwegian (69.9%). *Post-hoc* tests (Bonferroni-corrected for multiple comparisons) revealed that English and Norwegian did not differ from one another (*p* = 1.0), but both differed from each of the other three languages (*p* < 0.001), and that German, Chinese, and Japanese did not differ from one another (*p* > 0.103).

The consistency values indicate that in general, the individual participant responded in a quite uniform manner, but these values also leave room for variation across samples (particularly for English and Norwegian), across the different types of tasks (Mov, Ord, vs. Ass), and across the adopted front assignment (to the smallest vs. the largest number). In the final step, we therefore inspected individual response patterns in order to qualify this variation. Do the responses attest to uniform, task-specific, or item-specific front assignments?

*(3) Individual response patterns*. This analysis was restricted to those participants who solved all five numerical items. First, we identified participants with a *uniform* front assignment either to the smallest or the largest number of a sequence across the five items. The remaining participants were then checked for *task-specific* response patterns. We determined whether or not the two items of the Moving Task were solved uniformly and whether or not the two items of the Order Task were solved uniformly, by assigning front either to the smallest or to the largest number. Cases with inconsistent front assignments constitute *item-specific* response patterns. The Front Assignment Task was not considered here as it consists of only one item of this type and hence precludes a distinction between task-specific and item-specific responses. The results are presented in Table 2.

**Table 2:** Individual response patterns across the five numerical items (in %, with respective *N* given in brackets).

	**English (*N* = 59)**	**Norwegian (*N* = 76)**	**German (*N* = 113)**	**Chinese (*N* = 87)**	**Japanese (*N* = 120)**
**UNIFORM** **F****ront** **ASSIGNMENT ACROSS THE THREE TYPES OF TASKS**					
Front=smallest	15.3 (9)	11.8 (9)	68.1 (77)	75.9 (66)	56.7 (68)
Front=largest	1.7 (1)	—	—	—	—
**TASK-SPECIFIC UNIFORM** **F****ront** **ASSIGNMENTS**					
**Moving Task**					
Front=smallest	28.8 (17)	14.5 (11)	16.8 (19)	17.2 (15)	27.5 (33)
Front=largest	27.1 (16)	44.7 (34)	3.5 (4)	1.1 (1)	0.8 (1)
*Not uniform*	*27.1 (16)*	*28.9 (22)*	*11.5 (13)*	*5.7 (5)*	*15.0 (18)*
**Order Task**					
Front=smallest	28.8 (17)	55.3 (42)	20.4 (23)	24.1 (21)	15.8 (19)
Front=largest	16.9 (10)	15.8 (12)	1.8 (2)	—	1.7 (2)
*Not uniform*	*37.3 (22)*	*17.1 (13)*	*9.7 (11)*	—	*25.8 (31)*
*M*_task-*specific F*ront_	50.8	65.1	21.3	21.3	22.9
*M_*item*-*specific F*ront_*	*32.2*	*23.0*	*10.6*	*2.9*	*20.4*

*Item-specific response patterns are set in italics*.

In line with the results from the item-level and consistency analyses, a strong difference emerged between German, Chinese, and Japanese on the one hand, and English and Norwegian on the other.

The majority of the German, Chinese, and Japanese participants assigned front uniformly across all items, and always to the smallest number (ranging from 56.7% for Japanese to 75.9% for Chinese). The mean proportion of task-specific front assignments was about 20%, with front assigned to the smallest number being the most frequent task-specific response. This finding indicates that task-specificity in this case does not result from differences in responses, but rather from uniform responses in one task combined with item-specific responses in the other. Finally, the mean proportion of item-specific responses was relatively low (ranging from 2.9% for Chinese to 20.4% for Japanese).

For English and Norwegian, the patterns are quite different. Uniform front assignments across all items were rather infrequent (17.0% for English; 11.8% for Norwegian); in all cases except one, front was again assigned to the smallest number. Instead, the mean proportion of task-specific front assignments was high (50.8% for English; 65.1% for Norwegian). Compared to the other three languages, front was more often assigned to the largest number; in fact, this was the modal response for the Moving Task in the Norwegian sample. Finally, the mean proportion of item-specific responses was higher for English and Norwegian (32.2 and 23.0%, respectively) than for the other three languages.

#### Front assignment on alphabetical and textual items

As for the numerical items, we begin with the item-level analyses (first for the alphabetical items, and then for the textual items), before determining participants' consistency in assigning front and their individual response patterns across items.

*(1) Item-level analyses*. The log-linear analyses indicated a strong main effect *language* for each of the eight alphabetical and textual items, and a modulating effect of how the items were phrased for six items. Participants' front assignments depending on language and phrasing are reported in Table 3.

**Table 3:** Percentage of (*N*) participants assigning Front to the beginning of a sequence for the four alphabetical items (α1–α4) and four textual items (s1, s2, w, q), depending on language and phrasing.

		**English**		**Norwegian**		**German**		**Chinese**		**Japanese**	
		**Phrasing**		**Phrasing**		**Phrasing**		**Phrasing**		**Phrasing**	
		**Forward**	**Backward**	**Forward**	**Backward**	**Forward**	**Backward**	**Forward**	**Backward**	**Forward**	**Backward**
**MOVING TASK**											
Mov_α1	Front=beginning^a^^,^^b^	56.7 (30)	35.5 (31)	41.0 (39)	42.1 (38)	80.0 (55)	86.2 (58)	95.5 (44)	100 (45)	87.2 (47)	100 (57)
Mov_s1	Front=beginning^a^^,^^c^	22.6 (31)	35.5 (31)	28.9 (38)	23.1 (39)	84.2 (57)	89.7 (58)	97.8 (45)	93.2 (44)	98.0 (51)	84.8 (46)
		**Phrasing**		**Phrasing**		**Phrasing**		**Phrasing**		**Phrasing**	
		**In front of**	**Behind**	**In front of**	**Behind**	**In front of**	**Behind**	**In front of**	**Behind**	**In front of**	**Behind**
**ORDER TASK**											
Ord_α2	Front=beginning^a^^,^^b^	80.6 (31)	61.3 (31)	87.5 (40)	84.2 (38)	100 (58)	96.6 (58)	100 (44)	100 (45)	82.0 (61)	91.9 (62)
Ord_α3	Front=beginning^a^^,^^b^	80.0 (30)	50.0 (30)	94.6 (37)	82.9 (41)	98.3 (58)	100 (58)	100 (44)	100 (45)	98.4 (61)	95.2 (62)
Ord_s2	Front=beginning^a^^,^^c^	83.3 (30)	61.3 (31)	93.1 (29)	61.3 (31)	100 (54)	96.6 (58)	97.8 (45)	97.7 (44)	75.0 (4)^f^	92.3 (13)^f^
		**Phrasing**		**Phrasing**		**Phrasing**		**Phrasing**		**Phrasing**	
		**F****ront**	**B****ack**	**F****ront**	**B****ack**	**F****ront**	**B****ack**	**F****ront**	**B****ack**	**F****ront**	**B****ack**
**F****ront** **ASSIGNMENT TASK**											
Ass_α4	Front=beginning^b^	93.3 (30)	59.4 (32)	68.3 (41)	30.6 (36)	98.3 (58)	93.1 (58)	100 (45)	97.7 (44)	88.7 (62)	90.2 (61)
	Front=end^b^	0.0	15.6	0.0	2.8	0.0	1.7	0.0	2.3	3.2	0.0
	Does not exist	6.7	25.0	29.3	61.1	1.7	5.2	0.0	0.0	8.1	4.9
	Other	0.0	0.0	2.4	5.6	0.0	0.0	0.0	0.0	0.0	4.9
Ass_w	Front=beginning^d^	90.0 (30)	50.0 (32)	56.1 (41)	18.9 (37)	98.3 (58)	93.1 (58)	95.6 (45)	97.7 (44)	75.8 (62)	86.9 (61)
	Front=end^d^	0.0	18.8	0.0	16.2	0.0	0.0	0.0	0.0	4.8	0.0
	Does not exist	10.0	31.2	43.9	40.5	1.7	5.2	4.4	0.0	14.5	13.1
	Other	0.0	0.0	0.0	24.3	0.0	1.7	0.0	2.3	4.8	0.0
Ass_q	Front=beginning^e^	96.7 (30)	78.1 (32)	95.0 (40)	73.0 (37)	93.1 (58)	94.8 (58)	97.8 (45)	100 (44)	74.2 (62)	73.8 (61)
	Front=end^e^	0.0	9.4	5.0	5.4	0.0	1.7	0.0	0.0	1.6	3.3
	Does not exist	3.3	9.4	0.0	0.0	1.7	0.0	0.0	0.0	19.4	19.7
	Other	0.0	3.1	0.0	21.6	5.2	3.4	2.2	0.0	4.8	3.3

a *Percentage Front=end is 100 – percentage Front=beginning*.

b *Beginning: first letter of the alphabet (“A”); end: last letter (“Z”)*.

c *Beginning: first word of a sentence; end: last word (read from left to right)*.

d *Beginning: first letter of a word; end: last letter (read from left to right)*.

e *Beginning: introduction part of a questionnaire; end: thanking part*.

f *The responses of many Japanese participants could not be coded properly due to an ambiguity in the Japanese version of this item*.

For the item *Mov_*α*1*, the analysis revealed two significant effects: a main effect *language* (*G*^2^[4] = 129.663; *p* < 0.001) and a small but significant interaction *language* × *phrasing* (*G*^2^[4] = 15.765; *p* = 0.003). Assignment of front to the beginning of the alphabet was frequent among speakers of German (83.2%), Chinese (97.8), and Japanese (94.2%), and substantially less frequent among speakers of English (45.9%) and Norwegian (41.6%). The interaction reflected differences between the two phrasings, mainly for English and Japanese. For English, assignment of front to the beginning of the alphabet was more frequent when the item asked about a letter being moved “forward” (56.7%) than when it asked about a letter being moved “backward” (35.5%). The pattern was reversed for Japanese: Assignment of front to the beginning of the alphabet was less frequent when the item was phrased with “forward” (87.2%) than when it was phrased with “backward” (100%). For the other languages, the difference between the two phrasings was only marginal (≤6.2%).

For the item *Ord_*α*2*, the main effect *language* (*G*^2^[4] = 51.692; *p* < 0.001) was the only significant effect. Different from the item *Mov_*α*1*, front was preferably assigned to the beginning of the alphabet in all languages: highly frequently among speakers of German (98.3%), Chinese (100%), Japanese (87.0%), and Norwegian (85.9%), and less so but still frequently among speakers of English (71.0%).

For the item *Ord_*α*3*, the analysis revealed two significant main effects: *language* (*G*^2^[4] = 68.264; *p* < 0.001) and *phrasing* (*G*^2^[1] = 8.227; *p* = 0.004). Again, front was preferably assigned to the beginning of the alphabet in all languages: highly frequently among speakers of German (99.1%), Chinese (100%), Japanese (96.7%), and Norwegian (88.5%), and less so but still frequently among speakers of English (65.0%). Overall, this preference was stronger when the item asked participants to indicate whether a letter is “in front of” another letter (95.7%) compared to whether a letter is “behind” another letter (89.4%).

For the item *Ass_*α*4*, the analysis again revealed two significant main effects: *language* (*G*^2^[12] = 110.202; *p* < 0.001) and *phrasing* (*G*^2^[3] = 18.120; *p* < 0.001). Assignment of front to the beginning of the alphabet was highly frequent among speakers of German (95.7%), Chinese (98.9%), and Japanese (89.4%), less so but still frequent among speakers of English (75.8%), and least frequent among speakers of Norwegian (50.6%). Overall, this preference was stronger when the item asked participants to indicate the front of the alphabet (front=beginning: 90.3% front=end: 0.8%; does not exist: 8.5%; other: 0.4%) than when it asked participants to indicate the back of the alphabet (front=beginning: 78.8%; front=end: 3.5%; does not exist: 15.6%; other: 2.2%). As with number lists (cf. item *Ass_n5*), for some participants, the alphabet lacks a front or back. This response was given by some English-speaking participants (16.1%) and was particularly frequent among the Norwegian-speaking participants (44.2%).

For the item *Mov_s1*, the analysis revealed two significant effects: a main effect *language* (*G*^2^[4] = 190.755; *p* < 0.001) and a small but significant three-way interaction *language* × *phrasing* × *order* (*G*^2^[4] = 11.247; *p* = 0.024). Assignment of front to the beginning of a sentence was highly frequent among speakers of German (87.0%), Chinese (95.5%), and Japanese (91.8%), but rather infrequent among speakers of English (29.0%) and Norwegian (26.0%). The interaction indicated minor moderating effects of the phrasing and item order.

For the item *Ord_s2*, the analysis revealed two significant main effects: *language* (*G*^2^[4] = 44.824; *p* < 0.001) and *phrasing* (*G*^2^[1] = 10.934; *p* < 0.001). Different from the item *Mov_s1*, front was preferably assigned to the beginning of a sentence in all languages: highly frequently among speakers of German (98.2%), Chinese (97.8%), and Japanese (88.2%), and less so but still frequently among speakers of English (72.1%) and Norwegian (76.7%). Overall, this preference was stronger when the item asked participants to indicate whether a word is “in front of” another word (94.4%) compared to whether a word is “behind” another word (84.2%).

For the item *Ass_w*, the analysis revealed three significant effects: a main effect *language* (*G*^2^[12] = 126.132; *p* < 0.001), a main effect *phrasing* (*G*^2^[3] = 15.471; *p* = 0.001), and an interaction *language* × *phrasing* (*G*^2^[12] = 44.849; *p* < 0.001). Assignment of front to the beginning of a word was highly frequent among speakers of German (95.7%) and Chinese (96.6%), less so but still frequent among speakers of Japanese (81.3%) and English (69.4%), and least frequent among speakers of Norwegian (38.5%). Overall, this preference was stronger when the item asked participants to indicate the front of a word (front=beginning: 83.5%; front=end: 1.3%; does not exist: 14.0%; other: 1.3%) compared to the back of the word (front=beginning: 74.6%; front=end: 5.2%; does not exist: 15.5%; other: 4.7%), but this difference does not hold uniformly for all samples (in fact, it was reversed for Japanese), as indicated by the interaction. For some participants, words lack a front or back. This response was given by some speakers of Japanese (13.8%) and English (21.0%), and was particularly frequent among speakers of Norwegian (42.3%).

Finally, for the item *Ass_q*, the main effect *language* (*G*^2^[12] = 73.846; *p* < 0.001) was the only significant effect. Front was preferably assigned to the beginning of a questionnaire in all languages: highly frequently among speakers of German (94.0%), Chinese (98.9%), English (87.1%), and Norwegian (84.4%), and less so but still frequently among speakers of Japanese (74.0%). Different from all other items of the Front Assignment Task, the response option “Something like front/back does not exist” did not play a major role for most samples (≤6.5%), except for the Japanese speakers (19.5%).

On the whole, the data of the alphabetical and textual items revealed a quite uniform assignment of front to the beginning of the alphabet or text segment for German, Chinese, and Japanese, and more mixed assignments of front for English and Norwegian. As expected, the control variable *item order* did not have much of an influence. The different phrasings played a role for six of the eight items, suggesting that the assignment of front to the beginning of the alphabet or text segment was more pronounced when an item asked participants to indicate whether something is “in front of” or is the “front” of a reference entity, but the pattern was not completely homogeneous. Regarding the three types of tasks, the results were fairly homogeneous for German, Chinese, and Japanese, but not for English and Norwegian; there, the modal response switched from an assignment of front to the end of the alphabet or sentence in the Moving Task to an assignment of front to the beginning in the Order Task, and to an assignment of front to the beginning or to “Something like that does not exist” in the Front Assignment Task.

*(2) Individual consistency*. Consistency values across the eight alphabetical and textual items were calculated as described for the numerical items. Across the five samples, front was assigned to either the smallest or the largest number with a mean consistency of 85.0%. An analysis of variance indicated significant differences between the languages; *F*_(4, 463)_ = 89.087; *p* < 0.001; η^2^ = 0.435. Consistency was high for the speakers of German (94.4%), Chinese (98.2%), and Japanese (87.3%), and was lower for the speakers of English (69.7%) and Norwegian (64.5%). *Post-hoc* tests (Bonferroni-corrected for multiple comparisons) revealed that English and Norwegian did not differ from one another (*p* = 0.354), but both differed from each of the other three languages (*p* < 0.001); that German and Chinese did not differ from one another (*p* = 0.597), but both differed from each of the other three languages (*p* < 0.002); and that Japanese differed from all other languages (*p* < 0.002).

*(3) Individual response patterns*. As for the numerical items, this analysis was restricted to those participants who solved the relevant alphabetical and textual items, comprising seven items for Japanese (excluding the item Ord_s2 that could not be coded appropriately for most participants) and all eight items otherwise. First, we identified participants with a *uniform* front assignment either to the beginning or the end of the alphabet or text segment across the whole set of items. The remaining participants were then checked for *task-specific* response patterns. We determined whether or not each of the three types of tasks was solved uniformly, by assigning front either to the beginning or to the end: the Moving Task with two items, the Order Task with three items (Japanese: two items), and the Front Assignment Task with three items. Again, cases with inconsistent front assignments constitute *item-specific* response patterns. The results are presented in Table 4.

**Table 4:** Individual response patterns across the eight (seven for Japanese^a^) alphabetical and textual items (in %, with respective *N* given in brackets).

	**English (*N* = 59)**	**Norwegian (*N* = 58)**	**German (*N* = 109)**	**Chinese (*N* = 89)**	**Japanese (*N* = 88)**
**UNIFORM** **F****ront** **ASSIGNMENT ACROSS THE THREE TYPES OF TASKS**					
Front=beginning	11.9 (7)	12.1 (7)	73.4 (80)	85.4 (76)	52.3 (46)
Front=end	—	—	—	—	—
**TASK-SPECIFIC UNIFORM** **F****ront** **ASSIGNMENT**					
**Moving Task**					
Front=beginning	13.6 (8)	12.1 (7)	3.7 (4)	7.9 (7)	35.2 (31)
Front=end	50.8 (30)	48.3 (28)	7.3 (8)	—	2.3 (2)
*Not uniform*	*23.7 (14)*	*27.6 (16)*	*15.6 (17)*	*6.7 (6)*	*10.2 (9)*
**Order Task**					
Front=beginning	32.2 (19)	53.4 (31)	23.9 (26)	12.4 (11)	36.4 (32)
Front=end	8.5 (5)	1.7 (1)	—	—	—
*Not uniform*	*47.5 (28)*	*32.8 (19)*	*2.8 (3)*	*2.4 (2)*	*11.4 (10)*
**F****ront** **Assignment Task**					
Front=beginning	54.2 (32)	15.5 (9)	19.3 (21)	9.0 (8)	14.8 (13)
Front=end	1.7 (1)	—	—	—	—
Does not exist	3.4 (2)	—	0.9 (1)	—	3.4 (3)
Other	—	—	—	—	—
*Not uniform*	*28.8 (17)*	*72.4 (42)*	*6.4 (7)*	*5.6 (5)*	*29.5 (26)*
*M*_task-*specific *F*ront*_	54.8	43.7	18.3	9.7	30.7
*M*_*task*-*specific *F*ront*_	*33.3*	*44.3*	*8.3*	*4.9*	*17.0*

*Item-specific response patterns are set in italics*.

a *In Japanese, the analysis is based on seven items only; the item Ord_s2 was excluded, because it was solved appropriately only by a handful of participants*.

In line with the previous results, a strong difference again emerged between German, Chinese, and Japanese on the one hand, and English and Norwegian on the other.

The majority of the German, Chinese, and Japanese participants assigned front uniformly across all items, and always to the beginning of the alphabet or text segment (ranging from 52.3% for Japanese to 85.4% for Chinese). The mean proportion of task-specific front assignments ranges from 9.7% for Chinese to 30.7% for Japanese, with front assigned to the beginning being the most frequent task-specific response. This finding again indicates that task-specificity in this case does not result from differences in responses, but rather from uniform responses in one task combined with item-specific responses in the other. Finally, the mean proportion of item-specific responses was relatively low (ranging from 4.9% for Chinese to 17.0% for Japanese).

For English and Norwegian, the patterns are quite different. Uniform front assignments across all items were again rather infrequent (11.9% for English and 12.1% for Norwegian); in all cases, front was again assigned to the beginning of the alphabet and text segment. The mean proportion of task-specific front assignments was high (54.8% for English; 43.7% for Norwegian). Compared to the other three languages, front was more often assigned to the end of the alphabet and text segment; in fact, this was the modal response for the Moving Task both in the English and the Norwegian sample. Finally, the mean proportion of item-specific responses was higher for English and Norwegian (33.3 and 44.3%, respectively) than for the other three languages. The high proportion of item-specific responses for Norwegian was mainly due to the Front Assignment Task, which showed a particularly high value (72.4%).

## Discussion

The main goal of the current study was to explore the potential for variability in spatial representations of number. Specifically, it aimed at investigating the extent to which such representations depend (i) on the perspective taken and other specifics of the tasks, (ii) on the linguistic and cultural background of participants, and (iii) on the research paradigm. While our findings so far paint a rather complex picture, they suggest that the spatial alignment of number representations is indeed more variable than previously assumed, and that all of the factors investigated do affect the alignment. In the following, we first outline and discuss (in reverse order) the emerging patterns for each factor in the numerical tasks, before comparing respective patterns across domains, both with the alphabetical and textual tasks reported above and with similar sets of tasks in the temporal and spatial domain as reported elsewhere.

### Sources of within-domain variability in the numerical task

Possible sources of the variability in numerical tasks include the research paradigm, cultural and linguistic differences, as well as the perspective chosen and other task specifics.

#### Research paradigm

Number representations, if spatialized in a linear manner, may unfold along three distinct dimensions: lateral (i.e., left/right), sagittal (back/front), or vertical (bottom/top), in either direction. Whereas standard paradigms for number line assessment predefine a particular spatial dimension as part of the task (e.g., SNARC tasks typically recruit the lateral dimension), and hence obtain spatial representations along this dimension, the current study used a language-based paradigm to probe whether a different (i.e., the sagittal) dimension may also be recruited for alignment. The findings from the current study indicate that this is indeed the case.

Specifically, numbers may be aligned not only with the *lateral* axis in either direction, as for speakers of the Germanic languages (Dehaene et al., 1993; Siegler and Opfer, 2003; Wood et al., 2008; Moeller et al., 2009), or with the *vertical* axis, as for speakers of Chinese and Japanese (Ito and Hatta, 2004; Hung et al., 2008), but also along the *sagittal* axis. Speakers of German, Chinese, and Japanese exhibited a strong preference for representing smaller numbers “in front of” larger numbers. This preference was less consistent, but nevertheless also observed, among the English and Norwegian speakers. Evidently, alignment of the number line with the sagittal axis makes sense for most of our participants (for evidence on a similar near-to-far alignment (see Santens and Gevers, 2008).

These findings imply not only that the spatial alignment of the number line may be more diverse than previously assumed, but also—and importantly—that people are prepared to adopt more than one such type of spatialized representation depending on the nature of the task context (see also Hung et al., 2008; Fischer et al., 2010; Winter et al., 2015). A possibility not explicitly tested in the current study, but raised by the parallels between number and time representations, is that distinct ways of anchoring (e.g., in the person him/herself or in external reference points) may also affect how the number line is spatialized (cf. Bender and Beller, 2014). This would also necessitate differentiating more strictly the dimensions under scrutiny and paying more diligence to how they are implemented in the experimental design. When, for instance, the sagittal axis (front/back) is conflated with a radial axis (near/far), or vertical representations are measured with tabletop layouts (i.e., along the sagittal/radial axis), findings and their interpretation are unnecessarily obscured (Winter et al., 2015).

#### Cultural and linguistic differences

Whereas most previous studies interested in the potential of cultural influences focused on the direction of reading and writing as the most obvious factor for shaping the MNL (e.g., Dehaene et al., 1993; Zebian, 2005; Shaki et al., 2009; for a more nuanced perspective see Shaki and Fischer, 2008, 2018; Fischer et al., 2010), we investigated whether native language and/or cultural background may also influence the MNL by other means. The findings reported above seem to confirm this, but inferences so far remain speculative.

Specifically, we did find significant cultural differences, but interestingly not along the lines one may have expected. While speakers of German, Chinese, and Japanese—three entirely unrelated languages—exhibited the same strong preference for the same type of representation (front pointing toward the smallest number), speakers of English and Norwegian—two close relatives of German—differed both from German and from each other. Notably, these differences emerged not so much in terms of different preferences for MNL orientation, but rather in an apparent overall lack of clear preferences on the part of English and Norwegian speakers. That is, within these two groups, not even within-cultural consensus was achieved. While the present findings cannot account for this lack of within-cultural consensus in MNL orientation, it is in line with a similar lack of consensus in MTL orientation for the same populations (Rothe-Wulf et al., 2015; Bender et al., 2017)—a pattern we will come back to in the section below in which we compare patterns across domains.

Since speakers of English, Norwegian, and German share almost identical writing systems—in contrast to Chinese and Japanese speakers—writing and reading direction can be excluded as a relevant factor for the differences observed here. The same is true for a possible influence of the counting system, especially in terms of the transparency and regularity in number word construction and of the patterns of finger counting, which were alternatively discussed as prime factors in shaping spatialized number lines (cf., Bender and Beller, 2011, 2018; Fischer and Brugger, 2011), as these differ substantially between German, Chinese, and Japanese. Which cultural (or other) factors may be responsible for these differences, then, remains unclear.

#### Perspective and other task specifics

Whereas a widespread assumption holds a homogeneous concept of the MNL as something rather stable and independent of the perspective taken, research on the domains of space and time points to the possibility that representations and inferences may change according to whether a superordinate field, a given reference point, or a subjective viewpoint is taken as the underlying frame of reference, and according to whether static or dynamic relations are at stake. To examine the potential influence of these factors, we therefore collected data on fixed (static) versus changing (dynamic) relations between specified numbers and number sequences, and on whether a spatial orientation can be assigned to number sequences and the number line itself as the superordinate field. For reasons of control, we also varied the polarity of the spatial expression under scrutiny, that is, whether items were phrased using the formulations “front,” “in front of,” and “forward,” or the reversed set “back,” “behind,” and “backward.”

Somewhat unexpectedly, the specific formulation used (i.e., “front/in front of/forward” vs. “back/behind/backward”) had significant effects on response patterns, and for speakers of English almost reversed the trend across the types of tasks. While this apparently inconsistent usage of complementary poles is hard to account for in the context of our study, it is not an unusual observation (e.g., Grabowski and Weiß, 1996; Grabowski and Miller, 2000). Against this background, in the following, we only consider the results of those tasks that were formulated with “front,” “in front of,” or “forward.”

As detailed above, three of our groups held strong preferences regarding the orientation of the number line along the sagittal axis, namely with front pointing toward the smallest number. While their strong and consensual preference does not leave much space for variation, the two remaining groups were sensitive to task specifics, and the patterns observed suggest that the same distinctions as for space and time may also be decisive for how the number line is oriented.

Specifically, the more explicitly the tasks ask for front in these ordered sequences, the more English speakers indicate the smallest number as in front: most strongly when explicitly assigning front to the number line (Front Assignment Task), less so when assessing the order of a sequence (Order Task), and least when moving a number forward (Moving Task). A similar pattern emerges for speakers of Norwegian, except that, in the Assignment Task, a substantially greater number of participants (34% as compared to 10% among English speakers) rejects the notion that a number sequence may have a front (perhaps due to an infelicitous translation of “front”; cf. Bender et al., 2017). Still, almost all of those who do consider this notion sensible agree on where front would be: pointing toward the smallest number and the beginning of a sequence (with 0% pointing to the opposite end of the sequence for all task items except the “questionnaire”). More importantly, this general trend of an increase in front assignment to the smaller number with increasing explicitness leads to a reversal of preferences for the least explicit task (i.e., the Moving Task). Here, speakers of Norwegian actually assigned front more often to the larger numbers.

### Comparison of patterns across domains

**Figure 3:**
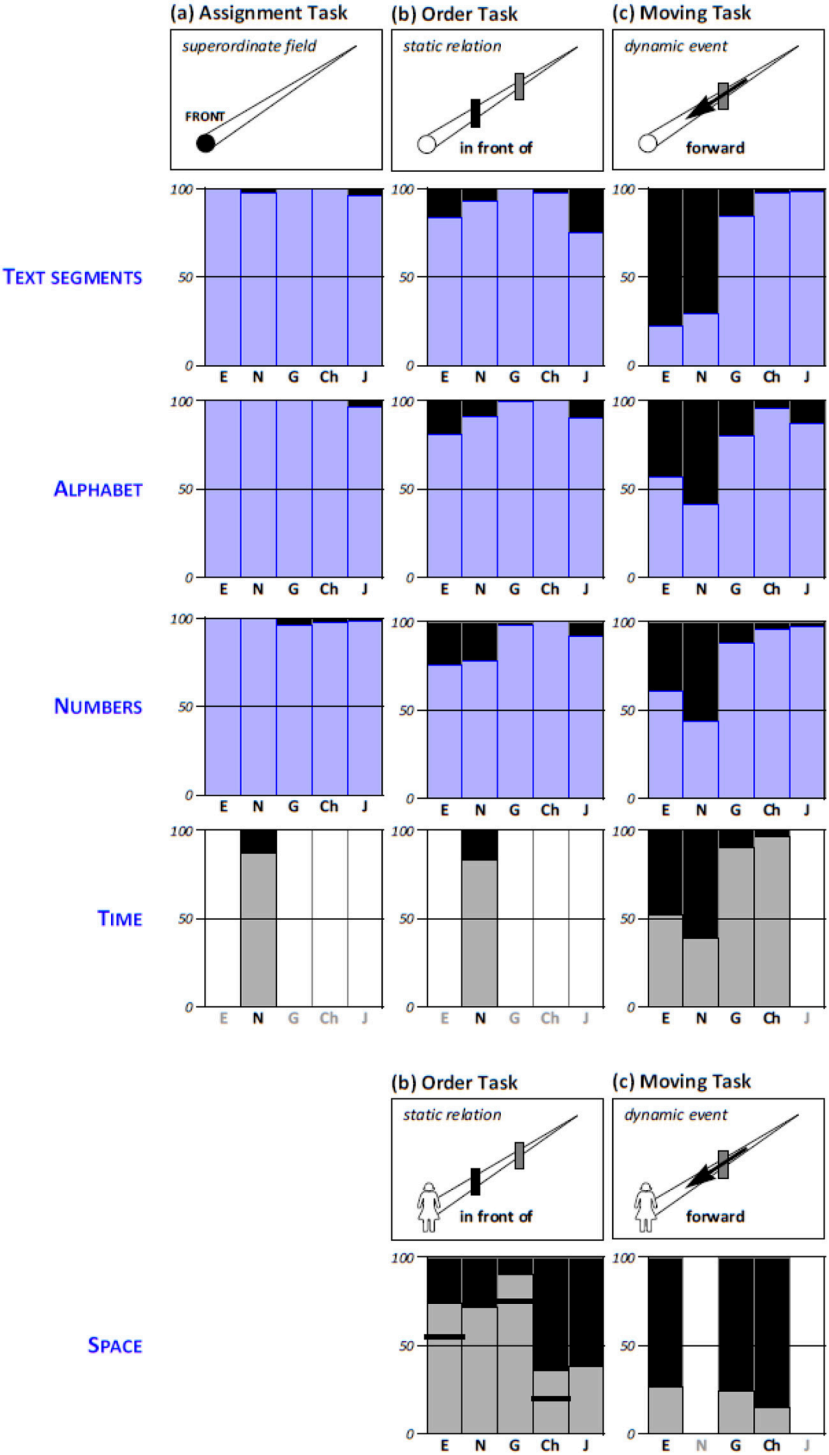
Response patterns across domains. The bars in the panels indicate the proportion of participants (in %) assigning front to the beginning of the ordered sequence (for text segments, the alphabet, number, or time) and toward the observer/Ego (for space). Data for the Assignment Tasks are recalculated to include only those who chose a specific direction; data for the textual and alphabetical items are aggregated across tasks, and data from the spatial domain are aggregated over reflection and rotation (which both imply the nearer item as in front, in contrast to translation). *Sources of additional data: Time* – Norwegian (Bender et al., 2017), English, German, and Chinese (Bender et al., 2010; see also Rothe-Wulf et al., 2015); *space/static* – English, German, and Chinese (Beller et al., 2015; see also Bender et al., 2012), Norwegian and Japanese (Beller and Bender, 2017); *space/dynamic* – English, German, and Chinese (Bender et al., 2012). The vertical strokes in the bars for space indicate the somewhat lower values for the static task as collected in the study that also investigated dynamic relations (Bender et al., 2012).

To investigate cross-domain correspondences, we compare the patterns of the numerical items first with those from the alphabetical and textual items reported above, and then with data on the temporal and spatial domain, obtained in related studies reported elsewhere. When comparing response patterns across domains, we consider all those items as in the same direction that—on a lateral axis—would be regarded as on the same side: that is, for instance, the smallest number, “a” in the Latin alphabet and the Japanese *kana*, the beginning of a piece of text, and—in the domain of time—the past (for speakers of the languages under scrutiny, all these directions would be localized left); for an overview (see Figure 3).

#### Comparison with the alphabetical and textual domain

Across the numerical, alphabetical, and textual domain, similarities in the response patterns are striking (see Figure 3). They are almost perfect for the Assignment Task, with the exception of the proportion to which participants chose the “does not exist” option, and for the Order Task. Patterns are also largely replicated for the Moving Task, yet with even lower assignments of front to the earlier items among speakers of English and Norwegian in the textual items as compared to the others. This coincides with a clear preference for the reversed orientation (i.e., later items as front) in the textual items, whereas the alphabetical and numerical items give rise to more ambivalence among speakers of these two languages.

#### Comparison with the temporal and spatial domain

To compare the data set reported here with data on the temporal and spatial domain, we draw on previously published findings (Bender et al., 2010, 2012, 2017; Beller et al., 2015; Beller and Bender, 2017). As some of these findings comprise partly different language selections, we lack comparable data for some of the languages in some of the tasks. Furthermore, for the spatial domain, two additional conventions need to be specified. First, a task corresponding to the Assignment Task used here is not possible for space as such because space has no beginning and, due to its greater number of dimensions, also has more degrees of freedom for alignment. Second, in order to establish comparable relations for the Order Task and the Moving Task, we pick those spatial items that contain a deictic center (i.e., an observer) as the component conveying orientation. In the spatial domain, such relations define a relative frame of reference in one of several variants (cf., Levinson, 2003); of the two variants relevant here, *reflection* renders the entity nearer to the observer as in front of the other, whereas *translation* renders the further-away entity as in front.

Interestingly, while the available temporal data—both from speakers of Norwegian obtained with the same set of tasks (Bender et al., 2017) and from speakers of English, German, and Chinese obtained with a different but structurally similar set of tasks (Bender et al., 2010; see also Rothe-Wulf et al., 2015)—closely reflect the numerical data, this does not hold for the spatial data (see Figure 3). In fact, the spatial response pattern is the one that most strikingly *differs* from the response patterns in all other domains. Here, the German pattern is closest to the pattern in the other two Germanic languages and is distinctly different from those in Chinese and Japanese^3^. Speakers of Chinese and Japanese exhibit an assignment pattern in the Order Task that is opposite to that in the numerical domain, albeit with a good deal of variability. And moving an entity forward strongly triggers front assignment to the further-away entity in all investigated languages alike.

### An account of cross-domain similarities and differences

As detailed in Figure 3, similarities across the domains investigated in the current study (i.e., number, alphabet, and text segments) as well as time are not perfect, but are substantial, for speakers of five different languages. Interestingly, of all domains, it is the spatial domain that does not fit the general pattern. What may explain both the convergence in the former and the disparity in the latter?

#### Ordered sequences

To illuminate what we hold to be the underlying mechanism, let us first return to the difference between tasks. All of the ordered sequences used here, including time, are conceived of as having a beginning: the Latin alphabet and the Japanese *kana* (according to the *gojuon* ordering) in the letter for “a,” the sequence of number words in 1, text segments in the first word written, and time in the past. At least metaphorically, beginning corresponds to front. This inherent orientation may also serve for ordering two elements within a sequence, localizing the earlier ones in the sequence as closer to its beginning. For instance, the smaller number, being closer to the front of the number sequence, would therefore be regarded as “in front of” the larger number. A dynamic context such as following the path of the sequence may activate a different perspective—one that shifts the assignment of front into the direction of the movement.

On this account, the Assignment Task should evoke an alignment of front with the beginning of the sequence across languages. In the Order Task, and even more so in the Moving Task, this preference may be superimposed to some extent by a preference for the reversed orientation, in that front is now more readily assigned to the direction of movement (as reflected in expressions like “counting forward/backward”). And indeed, in the Assignment Task, in which motion plays no role, the overwhelming majority of participants assign front to the beginning. In the Moving Task, this preference appears to come into conflict with the reversed preference for dynamic settings, which is why consensus is lowest here. Responses in the Order Task are interjacent. These assumptions are compatible with how, across languages and cultures, our participants responded to the tasks; cultural differences mainly emerged with regard to the extent to which the dynamic aspect triggered a reversal of perspectives (for a particularly striking case, see Rothe-Wulf et al., 2015).

This account offers an explanation not only for the convergence across cultures, but also for the convergence across domains. The reason for the latter, we propose, is that all domains—except for space—share important characteristics and may even be based on overlapping representations. For instance, the number sequence and the alphabet are organized in very similar ways, one arguably patterned on the other. Both are ordered sequences, recited endlessly for memorization in childhood, structurally similar to sentences and, when noted down, constituting a specific genre of text. All of these also share characteristics with time. On the one hand, time is generally organized by numbers, most obvious in how we specify date and time. On the other hand, ordered sequences such as numbers or letters also unfold along the temporal dimension: When enumerating the list of number words, reciting the letters of the alphabet, and writing sentences or larger pieces of text, the same process turns future or further-away entities into past and nearer entities. As we recite the sequence of counting words, for instance, it is the smaller ones that move further away into the past as time passes by.

Some empirical support comes from recent work by Sasanguie and colleagues. While taking an entirely different approach, their work confirms a central role of memory processes in the construction of symbolic number representations. Specifically, their findings point to the associations between numbers stored in long-term memory as a key factor for stable numerical representations and arithmetic competence (Sasanguie et al., 2017), thereby also supporting the critical shift from cardinal to ordinal processing in the development of children's numerical understanding (Sasanguie and Vos, 2018). This crucial role of verbal encoding for a linear spatial representation of serial order information is further emphasized by the difficulties of deaf individuals in recalling items in a given temporal sequence (reviewed in Rinaldi et al., 2018), but more research is needed to investigate whether this also affects the construction of a number line.

#### The case of space

Space is strikingly different. Not only does it have more dimensions than the other domains under scrutiny, but it also lacks inherent structure, order, and orientation. Apart from a single somewhat privileged direction, defined by gravitation, all other attempts of ordering presuppose a human perspective. Near versus far, front versus back, left versus right all depend on a subjective point of view, and even the non-relativistic reference points that define an absolute frame of reference such as cardinal directions, the slope of mountain sides, or a river's direction of flow require cultural conventions (Levinson, 2003; Bender and Beller, 2014). This may explain why, despite substantial consistency across other domains, response patterns in the spatial domain do not necessarily converge with any of the others.

#### Evolution of alignment patterns

If this account is valid, the mechanism that may have given rise to spatialized number lines would be less likely a result of a predisposition for a certain type of representations, and more likely a result of cultural evolution (Winter et al., 2015; Núñez, 2017), in the course of which a diverse set of cultural representations emerged that helped us put order into important domains. One of these representations was powerful enough to enable the alignment of ordered sequences across domains. Still, coming up with linear representations is far less trivial than we tend to believe. With the exception of rulers (that actually are an attempt to organize space by way of numbers, rather than the reverse), none of the cultural representations of number or time (and text) is strictly linear, or even linear at all. Number representations, for instance, at least in the decimal systems of the languages under scrutiny here, are 2-dimensional (Zhang and Norman, 1995), and even in the lower range in which they are still 1-dimensional, they are not necessarily represented in a line on a substantial number of cultural devices (e.g., telephone keypads and door locks). Likewise, the Japanese *kana* syllabaries are tabulated to begin with (Figures 2A,B), while the letters of the Latin alphabet in the medium with which we are arguably most frequently confronted are presented neither in a linear nor even an ordered manner (to verify, simply look at your computer's keyboard)—not to mention the innumerous combinations into which they are turned in daily life. Layout for most texts is also 2-dimensional, with lines running primarily left-to-right, but also top-to-bottom on a page. And even time is typically represented cyclically, emphasizing the recurrence of seconds, minutes, and hours (on analog clocks), or in a tabulated manner for both clock-time (on digital clocks) and larger units such as weekdays, months, or years (on calendars). None of these representations should actually prepare people to develop line-like representations, and both historical sources and data on synesthesia attest to some of the ensuing variability (e.g., Galton, 1883; Ernest, 1986; Bender and Beller, 2011; Núñez, 2011).

Arguably the only strictly linear—yet also entirely non-spatial—mode of representation in all domains discussed here (and across languages) is the verbal routine of reciting, be it for the alphabet or the sequence of counting words (the latter initially reinforced by finger counting; cf. Beller and Bender, 2011; Fischer and Brugger, 2011). Once in place, this linear sequence can be harnessed for organizing similarly structured domains such as loudness (Núñez et al., 2011) or time. Space, by contrast, with its three dimensions and lack of inherent structure, defies simple ways of sequencing. While some spatial arrangements do receive order with the help of one of the above domains, such as when hotel rooms or train cars are numbered, most arrangements in the spatial domain necessitate a rather complex coding based on a coordinate system for which both anchoring and aligning need further specification (Levinson, 2003; Bender and Beller, 2014). Taken together, this raises the question of whether it is really (the allegedly more concrete) space that serves as the universal foundation for representations of more abstract domains such as number and time, or whether inherent features of the latter two—as ordered sequences—are actually what facilitate our organization of space (for examples and other arguments why spatial representations of number or time may not be universal, see also Hutchins, 1983; Núñez, 2011, 2017; Núñez and Cornejo, 2012; Bender and Beller, 2014).

### Directions for future research

As discussed earlier, standard paradigms for number line assessment obtain spatial representations along the lateral dimension, because they predefine this dimension as part of the task. One of the most important achievements of the current study is therefore its use of a language elicitation task, which allows us to tap into a different (i.e., the sagittal) dimension. Admittedly, the set of tasks in our study also predefines a dimension, even if a different one, in either providing forced-choice response options (e.g., “Number 25 is two positions *in front of/behind* number 23”) or by phrasing the task itself using the dimension under scrutiny (e.g., “Which letter is directly *in front of/behind* G in the alphabet?”). Given our interest in establishing whether the sagittal axis can be used to represent numbers and the fact that such phrasings are more natural in language than left/right or top/down phrasings, we considered this approach justified for an exploratory study. However, if aiming for a more comprehensive understanding of how number representations may principally be aligned with space, future research would be well advised to open up the scope for possible responses. This should include an investigation of whether distinct ways of anchoring, if occurring at all, affect how the number line is spatialized.

A second way in which the current work should be extended lies in the range of languages investigated and the linguistic and cultural factors thus targeted. Specifically, while we attempted to include languages with different writing and reading directions, our selection does not cover the full range of variability in this regard. The same is true for finger counting patterns or properties of counting systems. With regard to the latter, for instance, users of body-based counting systems like the Oksapmin (Saxe, 1981) would be an informative sample. Including more characteristics of cultural groups and language communities may also help to answer the puzzling question of why speakers of closely related languages like the Germanic languages tend to differ so substantially in their mental representations of number and time lines. To this effect, other sample characteristics like differences between dialects or effects of bilingualism would be worth investigating.

In addition, research on people with sensory deprivation would be able to shed more light on which aspects of number representation are possibly innate, which are based on sensorimotor experience of movement, and which are brought about by cultural practices and linguistic routines. Unfortunately, while this line of research is experiencing an upsurge, studies devoted to numerical representations along the sagittal axis are still missing (Rinaldi et al., 2018).

A third possible direction for future research could be a more in-depth investigation of the potential role played by perspective and other task specifics. Apparently, the involvement of motion, for instance, has the potential to induce a perception of “forward” in the direction of larger numbers, in line with linguistic expressions like “counting forward.” Surprisingly, however, this perspective was observed only in two of the five groups, and even there it was not strong enough to fully reverse the preference for assigning front to the beginning of the number line in static relations. Exactly which factors contribute to the partial reversal of front assignment in some groups, but not in others, therefore remains an open question.

And finally, more research should be devoted to the analysis of changes over time. While we already discussed the impact that cultural and linguistic tools may have had on the emergence and evolution of number line representations, their influence on children's development deserve similar attention. Apparently, children's increasing understanding of numbers involves an increasing number of symbolic, culture-specific representations; as a result, the application of procedural knowledge is gradually replaced by the retrieval of declarative knowledge (Sasanguie and Vos, 2018). This raises the interesting question of whether and how increasing knowledge of other cultural systems (e.g., temporal representations or the alphabet) may affect how children learn to represent and process information from those domains, or whether and when generalizations across domains may emerge.

## Conclusion

Number lines and time lines are an appealing possibility compared to the many other ways in which numbers or dates may be mapped onto spatial representations. However, the high degree of variability in the dimensions or axes recruited and in the orientation of alignment with these axes suggests that no specific linear representation is exclusive or essential. Unless we open up our horizon for alternative possibilities, and amend our toolkit with alternative techniques and tasks, we will not be able to find out which possibilities for representing number, time, and other domains, beyond these simple lines, humans actually possess. People are highly flexible in their representations—and prepared to demonstrate this if only they are provided with respective opportunities. Future research should therefore take this more seriously, both with regard to their theoretical conceptualization and to the designs of research paradigms and tasks.

## Ethics statement

Although our university ethics board only deals with medical research, we can confirm that we follow the Frankfurt declaration of ethical conduct for anthropological research, which addresses all stages of the research project from designing to reporting the research.

## Author contributions

All authors listed have made substantial, direct and intellectual contribution to the work and approved it for publication.

### Conflict of interest statement

The authors declare that the research was conducted in the absence of any commercial or financial relationships that could be construed as a potential conflict of interest.

## Footnotes

^1^By accident, for the Japanese version of the item *Mov_n2*, only the forward movement was implemented in all versions of the questionnaire.

^2^*N* equals five numerical items minus the number of missing responses.

^3^Values for the static task were somewhat lower across the board in the study that also investigated dynamic relations (Bender et al., 2012). There, front was assigned to the nearer token by 56.5, 74.6, and 20.3% of the very same speakers of English, German, and Chinese, respectively (indicated in Figure 3 by the vertical stroke in the respective bars).

## References

[B1] Beller S. Bender A. (2011). Explicating numerical information: when and how fingers support (or hinder) number comprehension and handling. Front. Psychol. 2*:*214. 10.3389/fpsyg.2011.00214 21960977 PMC3178230

[B2] Beller S. Bender A. (2017). How relative is the relative frame of reference? Front and back in Norwegian, Farsi, German, and Japanese, in Proceedings of the 39th Annual Conference of the Cognitive Science Society, eds Gunzelmann G. Howes A. Tenbrink T. Davelaar E. J. (Austin, TX: Cognitive Science Society), 118–123.

[B3] Beller S. Bender A. Chrisomalis S. Jordan F. M. Overmann K. A. Saxe G. B. (2018). The cultural challenge in mathematical cognition. J. Numer. Cogn. 4, 448–463. 10.5964/jnc.v4i2.137

[B4] Beller S. Singmann H. Hüther L. Bender A. (2015). Turn around to have a look? Spatial referencing in dorsal vs. frontal settings in cross-linguistic comparison. Front. Psychol. 6*:*1283. 10.3389/fpsyg.2015.01283 26388802 PMC4556973

[B5] Bender A. Beller S. (2011). Cultural variation in numeration systems and their mapping onto the mental number line. J. Cross Cult. Psychol. 42, 579–597. 10.1177/0022022111406631

[B6] Bender A. Beller S. (2012). Nature and culture of finger counting: diversity and representational effects of an embodied cognitive tool. Cognition 124, 156–182. 10.1016/j.cognition.2012.05.005 22695379

[B7] Bender A. Beller S. (2014). Mapping spatial frames of reference onto time: a review of theoretical accounts and empirical findings. Cognition 132, 342–382. 10.1016/j.cognition.2014.03.016 24873738

[B8] Bender A. Beller S. (2018). Numeration systems as cultural tools for numerical cognition, in Mathematical Cognition and Learning: Language and Culture in Mathematical Cognition, eds Berch D. B. Geary D. C. Mann Koepke K. (Cambridge, MA: Academic Press), 297–320. 10.1016/B978-0-12-812574-8.00013-4

[B9] Bender A. Beller S. Bennardo G. (2010). Temporal frames of reference: conceptual analysis and empirical evidence from German, English, Mandarin Chinese, and Tongan. J. Cogn. Cult. 10, 283–307. 10.1163/156853710X531195

[B10] Bender A. Rothe-Wulf A. Hüther L. Beller S. (2012). Moving forward in space and time: how strong is the conceptual link between spatial and temporal frames of reference? Front. Psychol. 3:486. 10.3389/fpsyg.2012.00486 23162519 PMC3498962

[B11] Bender A. Sjåfjell K. Rothe-Wulf A. Beller S. (2017). Representing time in terms of space: directions of mental timelines in Norwegian, in Proceedings of the 39th Annual Conference of the Cognitive Science Society, eds Gunzelmann G. Howes A. Tenbrink T. Davelaar E. J. (Austin, TX: Cognitive Science Society), 1617–1622.

[B12] Bennardo G. (2009). Language, Space and Social Relationships: A Foundational Cultural Model in Polynesia. Cambridge: Cambridge University Press.

[B13] Bergen B. K. Chan Lau T. T. (2012). Writing direction affects how people map space onto time. Front. Psychol. 3*:*109. 10.3389/fpsyg.2012.00109 22514546 PMC3322406

[B14] Bonato M. Zorzi M. Umiltà C. (2012). When time is space: evidence for a mental time line. Neurosci. Biobehav. Rev. 36, 2257–2273. 10.1016/j.neubiorev.2012.08.007 22935777

[B15] Boroditsky L. (2000). Metaphoric structuring: understanding time through spatial metaphors. Cognition 75, 1–27. 10.1016/S0010-0277(99)00073-6 10815775

[B16] Boroditsky L. Gaby A. (2010). Remembrances of times east: absolute spatial representations of time in an Australian aboriginal community. Psychol. Sci. 21, 1635–1639. 10.1177/0956797610386621 20959511

[B17] Calude A. S. Verkerk A. (2016). The typology and diachrony of higher numerals in Indo-European: a phylogenetic comparative study. J. Lang. Evol. 1, 91–108. 10.1093/jole/lzw003

[B18] Casasanto D. Jasmin K. (2012). The hands of time: temporal gestures in English speakers. Cogn. Linguist. 23, 643–674. 10.1515/cog-2012-0020

[B19] Dehaene S. Bossini S. Giraux P. (1993). The mental representation of parity and number magnitude. J. Exp. Psychol. Gen. 122, 371–396. 10.1037/0096-3445.122.3.371

[B20] Dehaene S. Izard V. Spelke E. Pica P. (2008). Log or linear? distinct intuitions of the number scale in Western and Amazonian indigene cultures. Science 320, 1217–1220. 10.1126/science.1156540 18511690 PMC2610411

[B21] Ernest P. (1986). Mental number line images. Teach. Math. Appl. 5, 1–2. 10.1093/teamat/5.1.1

[B22] Fischer M. H. Brugger P. (2011). When digits help digits: spatial-numerical associations point to finger counting as prime example of embodied cognition. Front. Psychol. 2*:*260. 10.3389/fpsyg.2011.00260 22028696 PMC3198540

[B23] Fischer M. H. Fias M. H. (2005). Spatial representation of numbers, in Handbook of Mathematical Cognition, ed Campbell J. I. D. (New York, NY: Psychology Press), 43–54.

[B24] Fischer M. H. Mills R. A. Shaki S. (2010). How to cook a SNARC: number placement in text rapidly changes spatial–numerical associations. Brain Cogn. 72, 333–336. 10.1016/j.bandc.2009.10.010 19917517

[B25] Fuhrman O. McCormick K. Chen E. Jiang H. Shu D. Mao S.. (2011). How linguistic and cultural forces shape conceptions of time: english and Mandarin time in 3D. Cogn. Sci. 35, 1305–1328. 10.1111/j.1551-6709.2011.01193.x 21884222

[B26] Galton A. (2011). Time flies but space does not: limits to the spatialisation of time. J. Pragmat. 43, 695–703. 10.1016/j.pragma.2010.07.002

[B27] Galton F. (1883). Inquiries into Human Faculty and its Development. New York, NY: Macmillan.

[B28] Göbel S. M. Shaki S. Fischer M. H. (2011). The cultural number line: a review of cultural and linguistic influences on the development of number processing. J. Cross Cult. Psychol. 42, 543–565. 10.1177/0022022111406251

[B29] Grabowski J. Miller G. A. (2000). Factors affecting the use of dimensional prepositions in German and American English: object orientation, social context, and prepositional pattern. J. Psycholinguist. Res. 29, 517–553. 10.1023/A:1005124210205

[B30] Grabowski J. Weiß P. (1996). The prepositional inventory of languages: a factor that affects comprehension of spatial prepositions. Lang. Sci. 18, 19–35. 10.1016/0388-0001(96)00005-8

[B31] Haun D. B. Rapold C. J. Janzen G. Levinson S. C. (2011). Plasticity of human spatial cognition: spatial language and cognition covary across cultures. Cognition 119, 70–80. 10.1016/j.cognition.2010.12.009 21238953

[B32] Hung Y. H. Hung D. L. Tzeng O. J. Wu D. H. (2008). Flexible spatial mapping of different notations of numbers in Chinese readers. Cognition 106, 1441–1450. 10.1016/j.cognition.2007.04.017 17572403

[B33] Hutchins E. (1983). Understanding Micronesian navigation, in Mental Models, eds Gentner D. Stevens A. L. (Hillsdale, NJ: Lawrence Erlbaum), 191–225.

[B34] Ito Y. Hatta T. (2004). Spatial structure of quantitative representation of numbers: evidence from the SNARC effect. Mem. Cogn. 32, 662–673. 10.3758/BF03195857 15478760

[B35] Kennedy J. J. (1992). Analyzing Qualitative Data. New York, NY: Praeger.

[B36] Lakoff G. Johnson M. (1980). Metaphors we Live by. Chicago, IL: University of Chicago Press.

[B37] Le Guen O. Pool Balam L. I. (2012). No metaphorical timeline in gesture and cognition among Yucatec Mayas. Front. Psychol. 3*:*271. 10.3389/fpsyg.2012.00271 22908000 PMC3415701

[B38] León-Portilla M. (1990). Time and Reality in the Thought of the Maya. Oklahoma, OK: University of Oklahoma Press

[B39] Levinson S. C. (2003). Space in Language and Cognition. Cambridge: Cambridge University Press. 10.1017/CBO9780511613609

[B40] Majid A. Bowerman M. Kita S. Haun D. B. M. Levinson S. C. (2004). Can language restructure cognition? the case for space. Trends Cogn. Sci. 8, 108–114. 10.1016/j.tics.2004.01.003 15301750

[B41] Miles L. K. Tan L. Noble G. D. Lumsden J. Macrae C. N. (2011). Can a mind have two time lines? exploring space–time mapping in Mandarin and English speakers. Psychon. Bull. Rev. 18, 598–604. 10.3758/s13423-011-0068-y 21347879

[B42] Miura I. T. (1987). Mathematics achievement as a function of language. J. Educ. Psychol. 79, 79–82. 10.1037/0022-0663.79.1.79

[B43] Moeller K. Pixner S. Kaufmann L. Nuerk H. C. (2009). Children's early mental number line: logarithmic or decomposed linear? J. Exp. Child Psychol. 103, 503–515. 10.1016/j.jecp.2009.02.006 19328495

[B44] Núñez R. Cooperrider K. Wassmann J. (2012b). Number concepts without number lines in an indigenous group of Papua New Guinea. PLoS ONE 7:e35662. 10.1371/journal.pone.0035662 22558193 PMC3338449

[B45] Núñez R. E. (2008). Reading between the number lines. Science 321:1293 10.1126/science.321.5894.1293 18772414

[B46] Núñez R. E. (2011). No innate number line in the human brain. J. Cross Cult. Psychol. 42, 651–668. 10.1177/0022022111406097

[B47] Núñez R. E. (2017). Is there really an evolved capacity for number? Trends Cogn. Sci. 21, 409–424. 10.1016/j.tics.2017.03.005 28526128

[B48] Núñez R. Cooperrider K. (2013). The tangle of space and time in human cognition. Trends Cogn. Sci. 17, 220–229. 10.1016/j.tics.2013.03.008 23608363

[B49] Núñez R. Cooperrider K. Doan D. Wassmann J. (2012a). Contours of time: topographic construals of past, present, and future in the Yupno valley of Papua New Guinea. Cognition 124, 25–35. 10.1016/j.cognition.2012.03.007 22542697

[B50] Núñez R. E. Cornejo C. (2012). Facing the sunrise: cultural worldview underlying intrinsic-based encoding of absolute frames of reference in Aymara. Cogn. Sci. 36, 965–991. 10.1111/j.1551-6709.2012.01237.x 22417143

[B51] Núñez R. Doan D. Nikoulina A. (2011). Squeezing, striking, and vocalizing: is number representation fundamentally spatial? Cognition 120, 225–235. 10.1016/j.cognition.2011.05.001 21640338

[B52] Núñez R. E. Sweetser E. (2006). With the future behind them. Convergent evidence from Aymara language and gesture in the crosslinguistic comparison of spatial construals of time. Cogn. Sci. 30, 401–450. 10.1207/s15516709cog0000_62 21702821

[B53] Rinaldi L. Merabet L. B. Vecchi T. Cattaneo Z. (2018). The spatial representation of number, time, and serial order following sensory deprivation: a systematic review. Neurosci. Biobehav. Rev. 90, 371–380. 10.1016/j.neubiorev.2018.04.021 29746876

[B54] Rothe-Wulf A. Beller S. Bender A. (2015). Temporal frames of reference in three Germanic languages: individual consistency, interindividual consensus, and cross-linguistic variability. Q. J. Exp. Psychol. 68, 917–939. 10.1080/17470218.2014.970205 25403820

[B55] Santens S. Gevers W. (2008). The SNARC effect does not imply a mental number line. Cognition 108, 263–270. 10.1016/j.cognition.2008.01.002 18313655

[B56] Sasanguie D. Lyons I. M. De Smedt B. Reynvoet B. (2017). Unpacking symbolic number comparison and its relation with arithmetic in adults. Cognition 165, 26–38. 10.1016/j.cognition.2017.04.007 28460351

[B57] Sasanguie D. Vos H. (2018). About why there is a shift from cardinal to ordinal processing in the association with arithmetic between first and second grade. Dev. Sci. 21:e12653. 10.1111/desc.12653 29417697

[B58] Saxe G. B. (1981). Body parts as numerals: a developmental analysis of numeration among the Oksapmin in Papua New Guinea. Child Dev. 52, 306–316. 10.2307/1129244

[B59] Senft G. (1997). Referring to Space, Edn. Oxford: Clarendon.

[B60] Shaki S. Fischer M. H. (2008). Reading space into numbers–a cross-linguistic comparison of the SNARC effect. Cognition 108, 590–599. 10.1016/j.cognition.2008.04.001 18514179

[B61] Shaki S. Fischer M. H. (2018). Deconstructing spatial-numerical associations. Cognition 175, 109–113. 10.1016/j.cognition.2018.02.022 29500963

[B62] Shaki S. Fischer M. H. Petrusic W. M. (2009). Reading habits for both words and numbers contribute to the SNARC effect. Psychon. Bull. Rev. 16, 328–331. 10.3758/PBR.16.2.328 19293102

[B63] Siegler R. S. Opfer J. E. (2003). The development of numerical estimation: evidence for multiple representations of numerical quantity. Psychol. Sci. 14, 237–250. 10.1111/1467-9280.02438 12741747

[B64] Sinha C. Da Silva Sinha V. Zinken J. Sampaio W. (2011). When time is not space: the social and linguistic construction of time intervals and temporal event relations in an Amazonian culture. Lang. Cogn. 3, 137–169. 10.1515/langcog.2011.006

[B65] Torralbo A. Santiago J. Lupiáñez J. (2006). Flexible conceptual projection of time onto spatial frames of reference. Cogn. Sci. 30, 745–757. 10.1207/s15516709cog0000_67 21702834

[B66] Tversky B. Kugelmass S. Winter A. (1991). Cross-cultural and developmental trends in graphic productions. Cogn. Psychol. 23, 515–557. 10.1016/0010-0285(91)90005-9

[B67] Walsh V. (2003). A theory of magnitude: common cortical metrics of time, space and quantity. Trends Cogn. Sci. 7, 483–488. 10.1016/j.tics.2003.09.002 14585444

[B68] Winter B. Matlock T. Shaki S. Fischer M. H. (2015). Mental number space in three dimensions. Neurosci. Biobehav. Rev. 57, 209–219. 10.1016/j.neubiorev.2015.09.005 26365108

[B69] Wood G. Willmes K. Nuerk H. C. Fischer M. H. (2008). On the cognitive link between space and number: a meta-analysis of the SNARC effect. Psychol. Sci. 50, 489–525. Available online at: http://psycnet.apa.org/record/2009-00781-003

[B70] Zebian S. (2005). Linkages between number concepts, spatial thinking, and directionality of writing: the snarc effect and the reverse snarc effect in English and Arabic monoliterates, biliterates, and illiterate Arabic speakers. J. Cogn. Cult. 5, 165–190. 10.1163/1568537054068660

[B71] Zhang J. Norman D. A. (1995). A representational analysis of numeration systems. Cognition 57, 271–295. 10.1016/0010-0277(95)00674-3 8556844

