# Supplementary material for: Variability in the Alignment of Number and Space Across Languages and Tasks
Source: Front Psychol. 2018 Oct 4;9:1724. doi: 10.3389/fpsyg.2018.01724 (PMC6180175; doi:10.3389/fpsyg.2018.01724)
Supplement: Supplementary file 1 [file Data_Sheet_1.pdf]

Supplementary Material

Variability in the Alignment of Number and Space Across Languages and Tasks

Andrea Bender[1,2,*], Annelie Rothe-Wulf[3], & Sieghard Beller[1,2]

[1] Department of Psychosocial Science, University of Bergen, N-5020 Bergen, Norway
[2] SFF Centre for Early Sapiens Behaviour (SapienCE), University of Bergen, N-5020 Bergen, Norway
[3] Department of Psychology, Freiburg University, D-79085 Freiburg, Germany

* **Correspondence:** Andrea Bender, Department of Psychosocial Science,
University of Bergen, Postbox 7807, N-5020 Bergen, Norway.
email: andrea.bender@uib.no

1. Items and Instructions

For each language, we provide the instructions of the three tasks used in this study (Moving Task, Order Task, and FRONT Assignment Task), and of the items. The following abbreviations are used for the items: numbers (n), alphabet (α), sentence (s), word (w), and questionnaire (q).

Supplementary Table 1.1. Instruction and items of the Moving Task (*Mov*).

	English	*Norwegian*	*German*	*Chinese*	*Japanese*
Instruction	In this block, you are asked to respond to four different questions on shifting dates or positions. Please, write down for each question what you think where to the movement has occurred.	I denne bolken vil vi be deg om å vurdere datoer og andre posisjoner som har flyttet på seg. På hvert spørsmål, skriv ned retningen du mener bevegelsen har gått i.	In diesem Block werden Sie gebeten, auf verschiedene Fragen zu sich ändernden Terminen oder Positionen zu antworten. Bitte geben Sie für jede Frage an, wohin die Bewegung Ihrer Meinung nach erfolgt ist.	在该板块中，请您对下列关于更改日期或者方位的问题进行作答。我们想要了解的是，您认为以下各项更改是往哪个方向进行的。	このブロックでは、曜日や時間の変更についての4つの質問にお答えいただきます。それぞれの問題について、変更後の曜日や時間をお答えください
Mov_n1	The 7th signpost was moved {forward/backward} by two positions. Which number does the signpost now have?<br>Answer: ________________	Det sjuende veiskiltet ble flyttet fram to posisjoner. Hvilket nummer i rekken har det nå?<br>Svar: ________________	Der siebte Wegweiser wurde um zwei Positionen {nach vorne/nach hinten} verschoben. Welche Nummer hat der Wegweiser jetzt?<br>Antwort: ________________	第七个路牌{往前/往后} 推了两个位置。<br>现在该路牌位于第几个位置？<br>答: ________________	7番目に位置している標識が2つ分だけ{前に/後ろに}<br>動きました。最終的には何番目の位置にありますか？<br>答え: ________________
Mov_n2	Jenny wanted to marry on the 15th of August, but the date had to be moved {forward/ backward} by 7 days. On what date does the wedding now take place?<br>Answer: ________________	Jenny ville gifte seg den 15. august, men datoen måtte flyttes {fram/bakover} sju dager. På hvilken dato er bryllupet nå?<br>Svar: ________________	Jenny wollte am 15. August heiraten, aber der Termin wurde um 7 Tage {nach vorne/ nach hinten} verschoben. An welchem Tag findet die Hochzeit jetzt statt?<br>Antwort: ________________	珍妮想要在08月15日结婚，但该日期{往前/往后} 推了7天。现在珍妮的婚礼在哪一天举行？<br>答: ________________	ジェニーは8月15日に結婚したかったのですが、その7日<br>{前に/後ろに}しなければなりません。最終的に結婚式は何月何日に行われることになりますか？<br>答え: ________________

Mov_α1	If, in the English alphabet, the letter “E” were moved {forward/backward} by one position, between which two letters would it end up?<br>Answer: ________________	Hvis man flytter “E” i alfabetet{fram/bakover} en posisjon, i mellom hvilke bokstaver vil den da være?<br>Svar: __________________	Wenn im deutschen Alphabet der Buchstabe “E” um eine Position {nach vorne/nach hinten} verschoben wird, zwischen welchen zwei Buchstaben steht er dann?<br>Antwort: _______________	如果英文字母中的字母“E”{往前/往后}推一个位置，那么，它将位于哪两个字母的中间？<br>答: __________________	もしも、ひらがな50音の中の「い」の位置が1文字分だけ{前に/後ろに}動くとしたら最終的にどのひらがなの間になりますか？<br>答え: ___________________
Mov_s1	If, in this sentence, the word “apple” were moved {forward/backward} by three positions, between which two words would it end up?<br>Answer: ________________	I denne setningen, hvis ordet “eple” ble flyttet {fram/bakover} tre posisjoner, i mellom hvilke ord vil det da stå?<br>Svar: __________________	Wenn in diesem Satz das Wort “Apfel” um drei Positionen {nach vorne/nach hinten} geschoben wird, zwischen welchen zwei Worten steht es dann?<br>Antwort: _______________	如果这句话中的‘狗’一字{往前/往后}推三个位置，那么，该字将处于哪两个字的中间？<br>答: __________________	この文章中において「リンゴ」という単語が3文字分だけ{前に/後ろに}動きました。最終的にはどの文字とどの文字の間にありますか？<br>答え: ___________________

Supplementary Table 1.2. Instruction and items of the Order Task (*Ord*).

	English	*Norwegian*	*German*	*Chinese*	*Japanese*
Instruction	In this block, you are asked to respond to questions on dates, numbers and other items that usually occur in order. Please, indicate for each question, how you would describe the relationship between these items.	I denne bolken, vil vi be deg om å svare på spørsmål om datoer, tall og andre ting som vanligvis skjer i en rekkefølge. Hvordan vil du beskrive forholdet mellom disse komponentene?	In diesem Block werden Sie gebeten, Fragen zu Ereignissen zu beantworten, die normalerweise in einer bestimmten Reihenfolge stattfinden. Bitte geben Sie für jede Frage an, wie Sie die Beziehung zwischen diesen Ereignissen beschreiben würden.	在该板块中，请您对下列事件问题进行作答。这些事件通常按一定的顺序出现。我们想要了解的是，您将如何描述这些事件之间的关系。	このブロックでは、日付、数字、そして一定の順番に従って起こるその他のことについての質問にお答えいただきます。それぞれの問題について、対象となっている事物の関係性についてあなたがどのように描写するかお答えください。
Ord_n3	Number 25 is<br>two positions …<br>□ in front of<br>□ behind<br>… number 23.	Nummer 25 er<br>to posisjoner …<br>□ foran<br>□ bak<br>... nummer 23.	Die Zahl 25 ist<br>zwei Positionen …<br>□ vor<br>□ hinter<br>... der Zahl 23.	数字25位于数字23的<br>□ 前面<br>□ 后面<br>两个位置。	25という数字は23という数字の2つ…<br>□前<br>□後

Ord_n4	Which number is 5 positions {in front of/behind} 9?<br>Answer: ________________	Hvilket nummer er 5 posisjoner {foran/bak} 9?<br>Svar: __________________	Welche Zahl ist 5 Positionen {vor/hinter} 9?<br>Antwort: ________________	位于9{前面/后面}5位的是哪个数字？<br>答: ____________________	数字の9の5つ{前/後}の数字はなんですか？<br>答え: ____________________
Ord_α2	In the alphabet, the letter M is …<br>□ in front of<br>□ behind<br>… the letter P.	I alfabetet er bokstaven M …<br>□ foran<br>□ bak<br>... bokstaven P.	Im Alphabet ist der Buchstabe M …<br>□ vor<br>□ hinter<br>... dem Buchstaben P.	在英文字母表中，字母M位于字母P的<br>□ 前面<br>□ 后面	ひらがな50音において「は」は「ま」の<br>□前<br>□後
Ord_α3	Which letter is directly {in front of/behind} G in the alphabet?<br>Answer: ________________	Hvilken bokstav er direkte {foran/bak} G i alfabetet?<br>Svar: __________________	Welcher Buchstabe ist im Alphabet direkt {vor/hinter} "G"?<br>Antwort: ________________	在英文字母表中，直接位于"G"{前面/后面}的是哪个字母？<br>答: ____________________	ひらがな50音において「け」のすぐ{前/後ろ}のひらがなはなんですか？<br>答え: ____________________
Ord_s2	In this sentence, which word is two positions {in front of/behind} the underlined word "two"?<br>Answer: ________________	I denne setningen, hvilket ord er to posisjoner {foran/bak} det understrekede ordet "to"?<br>Svar: __________________	Welches Wort befindet sich in diesem Satz zwei Positionen {vor/hinter} dem unterstrichenen Wort "zwei"?<br>Antwort: ________________	这句话中的哪个字位于带下划线的"两"这个字的{前/后}两个位置？<br>答: ____________________	この文章中において、下線が引かれている2という数字の2つ{前/後}の文字はなんですか？<br>答え: ____________________

Supplementary Table 1.3. Instruction and items of the FRONT Assignment Task (*Ass*).

	English	*Norwegian*	*German*	*Chinese*	*Japanese*
Instruction	Please indicate for the following examples, where – according to your own personal feeling – {*front/back*} is.	Vennligst marker, i de følgende eksemplene, hvor du føler {*"forsiden"/"baksiden"*} er.	Bitte geben Sie bei den folgenden Beispielen an, wo nach Ihrem persönlichen Sprachgefühl {*vorne/hinten*} ist.	请阅读下列问题，根据您的语感回答，哪项属于{*前面/后面*}.	あなたの個人的な{「前方」/「後方」}の感覚に従って、以下の質問にお答えください。

Ass_n5	{*Front/Back*} of<br>an ordered number list …<br>□ is at the smallest number.<br>□ is at the largest number.<br>□ Something like this does not exist.<br>□ Something else, namely _______.	{*“Forsiden”/“baksiden”*} av en sortert tall-liste …<br>□ er ved det minste tallet.<br>□ er ved det største tallet.<br>□ Noe sånt finnes ikke.<br>□ Noe annet, nemlig: __________.	{*Vorne/Hinten*} bei einer geordneten Zahlenreihe ist …<br>□ bei der kleinsten Zahl.<br>□ bei der größten Zahl.<br>□ So etwas gibt es nicht.<br>□ etwas anderes, nämlich: ___________.	在一组有序的数字列中，属于{*前面/后面*}的是..<br>□ 最小的数字<br>□ 最大的数字<br>□ 没有此类现象<br>□ 其他，即: ___________。	番号順リストの{「前方」/「後方」}とは…<br>□ 最小の数<br>□ 最大の数<br>□ そのようなものは存在しない<br>□ その他、すなわち: _________
Ass_α4	{*Front/Back*} of<br>the English alphabet …<br>□ is at the letter “a”.<br>□ is at the letter “z”.<br>□ Something like this does not exist.<br>□ Something else, namely _______.	{*“Forsiden”/“baksiden”*} av det norske alfabetet …<br>□ er ved bokstaven “a”.<br>□ er ved bokstaven “å”.<br>□ Noe sånt finnes ikke.<br>□ Noe annet, nemlig: __________.	{*Vorne/Hinten*} im deutschen Alphabet ist …<br>□ der Buchstabe “a”.<br>□ der Buchstabe “z”.<br>□ So etwas gibt es nicht.<br>□ etwas anderes, nämlich: ___________.	在英文字母表中，属于{*前面/后面*}的是..<br>□ 字母“a”<br>□ 字母“z”<br>□ 没有此类现象<br>□ 其他，即: ___________。	ひらがな50音の{「前方」/「後方」}とは…<br>□「あ」<br>□「ん」<br>□ そのようなものは存在しない<br>□ その他、すなわち: _________
Ass_w	{*Front/Back*} of<br>the word “holiday” …<br>□ is at the letter “h”.<br>□ is at the letter “y”.<br>□ Something like this does not exist.<br>□ Something else, namely _______.	{*“Forsiden”/“baksiden”*} av ordet “ferie”…<br>□ er ved bokstaven “f”.<br>□ er ved bokstaven “e”.<br>□ Noe sånt finnes ikke.<br>□ Noe annet, nemlig: __________.	{*Vorne/Hinten*} in dem Wort “feiern” ist …<br>□ beim Buchstaben “f”.<br>□ beim Buchstaben “n”.<br>□ So etwas gibt es nicht.<br>□ etwas anderes, nämlich: ___________.	在“圣诞老人”这个词语中，属于{*前面/后面*}的是..<br>□ “圣”这个字<br>□ “人”这个字<br>□ 没有此类现象<br>□ 其他，即: ___________。	「きゅうじつ」という言葉の{「前方」/「後方」}とは…<br>□「き」の文字<br>□「つ」の文字<br>□ そのようなものは存在しない<br>□ その他、すなわち: _________
Ass_q	{*Front/Back*} of a questionnaire …<br>□ is at the instruction part.<br>□ is at the thanking part.<br>□ Something like this does not exist.<br>□ Something else, namely _______.	{*“Forsiden”/“baksiden”*} av et spørreskjema …<br>□ er ved instruksjonsdelen.<br>□ er ved takk for deltakelsen-delen.<br>□ Noe sånt finnes ikke.<br>□ Noe annet, nemlig: __________.	{*Vorne/Hinten*} bei einem Fragebogen ist …<br>□ die Instruktion.<br>□ die Danksagung für die Teilnahme.<br>□ So etwas gibt es nicht.<br>□ etwas anderes, nämlich: ___________.	在一份问卷中，属于{*前面/后面*}的是..<br>□ 说明<br>□ 对参与者的感谢<br>□ 没有此类现象<br>□ 其他，即: ___________。	アンケートの{「前方」/「後方」}とは…<br>□ 答え方に関する指示パート<br>□ 回答者へのお礼のパート<br>□ そのようなものは存在しない<br>□ その他、すなわち: _________

2. Item-Level Analyses

The following supplementary tables provide the results from the item-wise log-linear analyses on the re-coded responses as explained in the main article. Each analysis included three independent variables: *language* (English, Norwegian, German, Chinese, Japanese), *phrasing* (version 1 vs. version 2), and *order* of items (order 1 vs. order 2). The left side of each table provides the computed models and their fit values (in terms of the G^2 statistics). Each model is represented by its highest interaction term(s) given in brackets, which include(s) all lower-level interactions (if applicable) and the main effects of the individual factors. Main effects and interactions were tested for significance by *model comparisons*, which are provided on the right side of the table. The analysis started with the full model (7) that includes the main effects and interactions of all factors. Then, we simplified the model stepwise by excluding one model term at a time as the basis for the next comparison. We first excluded terms that involved the order of items, as we did not expect to find effects of this control variable, and then inspected effects of phrasing and language.

Supplementary Table 2.1. Log-linear analysis for the item Mov_n1 ("The 7th signpost was moved {forward/backward} by two positions. Which number does the signpost now have?").

Nr	*Model*	G^2	*df*	*p*	*Effect*	*Comparison*	ΔG^2	Δdf	*p*
(7)	(language × phrasing × order)	0	0	1					
(6)	(language × phrasing) & (language × order) & (phrasing × order)	9.038	4	.060	language × phrasing × order	(6) – (7)	9.038	4	.060
(5)	(language × phrasing) & (language × order)	9.149	5	.103	phrasing × order	(5) – (6)	0.111	1	.739
(4)	(language × phrasing) & order	10.336	9	.324	language × order	(4) – (5)	1.187	4	.880
(3)	(language × phrasing)	10.773	10	.375	order	(3) – (4)	0.437	1	.509
(2)	language & phrasing	19.659	14	.141	language × phrasing	(2) – (3)	8.886	4	.064
(1)	language	22.447	15	.097	phrasing	(1) – (2)	2.788	1	.095
(0)	*null-logit*	111.388	19	<.001	language	(0) – (1)	88.941	4	<.001

Supplementary Table 2.2. Log-linear analysis for the item Mov_n2 (“Jenny wanted to marry on the 15th of August, but the date had to be moved {forward/backward} by 7 days. On what date does the wedding now take place?”).

Nr	*Model*	G^2	*df*	*p*	*Effect*	*Comparison*	ΔG^2	Δdf	*p*
(7)	(language × phrasing × order)	0	0	1					
(6)	(language × phrasing) & (language × order) & (phrasing × order)	9.969	3	.019	language × phrasing × order	(6) – (7)	9.969	3	.019
(5)	(language × phrasing) & (language × order)	11.128	4	.025	phrasing × order	(5) – (6)	1.159	1	.282
(4)	(language × phrasing) & order	15.458	8	.051	language × order	(4) – (5)	4.330	4	.363
(3)	(language × phrasing)	15.695	9	.074	order	(3) – (4)	0.237	1	.626
(2)	language & phrasing	22.395	12	.033	language × phrasing	(2) – (3)	6.700	3	.082
(1)	language	22.412	13	.049	phrasing	(1) – (2)	0.017	1	.896
(0)	*null-logit*	176.822	17	<.001	language	(0) – (1)	154.410	4	<.001

Note. The degrees of freedom are reduced for some models and effects due to the fact that, in the Japanese questionnaire, the factor *phrasing* was accidentally implemented only in the version “forward”.

Supplementary Table 2.3. Log-linear analysis for the item Mov_α1 (“If, in the English alphabet, the letter “E” were moved {forward/backward} by one position, between which two letters would it end up?”).

Nr	*Model*	G^2	*df*	*p*	*Effect*	*Comparison*	ΔG^2	Δdf	*p*
(7)	(language × phrasing × order)	0	0	1					
(6)	(language × phrasing) & (language × order) & (phrasing × order)	3.953	4	.412	language × phrasing × order	(6) – (7)	3.953	4	.412
(5)	(language × phrasing) & (language × order)	4.164	5	.526	phrasing × order	(5) – (6)	0.211	1	.646
(4)	(language × phrasing) & order	9.844	9	.363	language × order	(4) – (5)	5.680	4	.224
(3)	(language × phrasing)	9.844	10	.454	order	(3) – (4)	<.001	1	.993
(2)	language & phrasing	25.609	14	.029	language × phrasing	(2) – (3)	15.765	4	.003
(1)	language	26.251	15	.035	phrasing	(1) – (2)	0.642	1	.423
(0)	*null-logit*	155.914	19	<.001	language	(0) – (1)	129.663	4	<.001

Supplementary Table 2.4. Log-linear analysis for the item Mov_s1 ("If, in this sentence, the word "apple" were moved {forward/backward} by three positions, between which two words would it end up?").

Nr	*Model*	G^2	*df*	*p*	*Effect*	*Comparison*	ΔG^2	Δdf	*p*
(7)	(language × phrasing × order)	0	0	1					
(6)	(language × phrasing) & (language × order) & (phrasing × order)	11.247	4	.024	language × phrasing × order	(6) – (7)	11.247	4	.024
(5)	(language × phrasing) & (language × order)	13.206	5	.022	phrasing × order	(5) – (6)	1.959	1	.162
(4)	(language × phrasing) & order	19.950	9	.018	language × order	(4) – (5)	6.744	4	.150
(3)	(language × phrasing)	20.258	10	.027	order	(3) – (4)	0.308	1	.579
(2)	language & phrasing	29.679	14	.008	language × phrasing	(2) – (3)	9.421	4	.051
(1)	language	29.928	15	.012	phrasing	(1) – (2)	0.249	1	.618
(0)	*null-logit*	220.683	19	<.001	language	(0) – (1)	190.755	4	<.001

Supplementary Table 2.5. Log-linear analysis for the item Ord_n3 ("Number 25 is two positions … □ in front of; □ behind …number 23.").

Nr	*Model*	G^2	*df*	*p*	*Effect*	*Comparison*	ΔG^2	Δdf	*p*
(7)	(language × phrasing × order)	0	0	1					
(6)	(language × phrasing) & (language × order) & (phrasing × order)	5.431	4	.246	language × phrasing × order	(6) – (7)	5.431	4	.246
(5)	(language × phrasing) & (language × order)	5.615	5	.346	phrasing × order	(5) – (6)	0.184	1	.668
(4)	(language × phrasing) & order	7.459	9	.589	language × order	(4) – (5)	1.844	4	.764
(3)	(language × phrasing)	8.209	10	.608	order	(3) – (4)	0.750	1	.386
(2)	language & phrasing	18.638	14	.179	language × phrasing	(2) – (3)	10.429	4	.034
(1)	language	20.169	15	.166	phrasing	(1) – (2)	1.531	1	.216
(0)	*null-logit*	81.807	19	<.001	language	(0) – (1)	61.638	4	<.001

Supplementary Table 2.6. Log-linear analysis for the item Ord_n4 (“Which number is 5 positions {in front of/behind} 9?”).

Nr	*Model*	G^2	*df*	*p*	*Effect*	*Comparison*	ΔG^2	Δdf	*p*
(7)	(language × phrasing × order)	0	0	1					
(6)	(language × phrasing) & (language × order) & (phrasing × order)	0.787	4	.940	language × phrasing × order	(6) – (7)	0.787	4	0.940
(5)	(language × phrasing) & (language × order)	2.448	5	.784	phrasing × order	(5) – (6)	1.661	1	0.197
(4)	(language × phrasing) & order	6.808	9	.657	language × order	(4) – (5)	4.360	4	0.359
(3)	(language × phrasing)	8.697	10	.561	order	(3) – (4)	1.889	1	0.169
(2)	language & phrasing	17.541	14	.229	language × phrasing	(2) – (3)	8.844	4	0.065
(1)	language	52.150	15	<.001	phrasing	(1) – (2)	34.609	1	<.001
(0)	*null-logit*	106.676	19	<.001	language	(0) – (1)	54.526	4	<.001

Supplementary Table 2.7. Log-linear analysis for the item Ord_α2 (“In the alphabet, the letter M is … □ in front of; □ behind … the letter P.”).

Nr	*Model*	G^2	*df*	*p*	*Effect*	*Comparison*	ΔG^2	Δdf	*p*
(7)	(language × phrasing × order)	0	0	1					
(6)	(language × phrasing) & (language × order) & (phrasing × order)	0.163	4	.997	language × phrasing × order	(6) – (7)	0.163	4	.997
(5)	(language × phrasing) & (language × order)	2.271	5	.811	phrasing × order	(5) – (6)	2.108	1	.147
(4)	(language × phrasing) & order	10.074	9	.345	language × order	(4) – (5)	7.803	4	.099
(3)	(language × phrasing)	10.200	10	.423	order	(3) – (4)	0.126	1	.723
(2)	language & phrasing	18.537	14	.183	language × phrasing	(2) – (3)	8.337	4	.080
(1)	language	18.798	15	.223	phrasing	(1) – (2)	0.261	1	.609
(0)	*null-logit*	70.490	19	<.001	language	(0) – (1)	51.692	4	<.001

Supplementary Table 2.8. Log-linear analysis for the item Ord_α3 (“Which letter is directly {in front of/behind} G in the alphabet?”).

Nr	*Model*	G^2	*df*	*p*	*Effect*	*Comparison*	ΔG^2	Δdf	*p*
(7)	(language × phrasing × order)	0	0	1					
(6)	(language × phrasing) & (language × order) & (phrasing × order)	2.828	4	.587	language × phrasing × order	(6) – (7)	2.828	4	.587
(5)	(language × phrasing) & (language × order)	4.898	5	.428	phrasing × order	(5) – (6)	2.070	1	.150
(4)	(language × phrasing) & order	9.044	9	.433	language × order	(4) – (5)	4.146	4	.387
(3)	(language × phrasing)	9.583	10	.478	order	(3) – (4)	0.539	1	.463
(2)	language & phrasing	12.629	14	.556	language × phrasing	(2) – (3)	3.046	4	.550
(1)	language	20.856	15	.141	phrasing	(1) – (2)	8.227	1	.004
(0)	*null-logit*	89.120	19	<.001	language	(0) – (1)	68.264	4	<.001

Supplementary Table 2.9. Log-linear analysis for the item Ord_s2 (“In this sentence, which word is two positions {in front of/behind} the underlined word “two”?”).

Nr	*Model*	G^2	*df*	*p*	*Effect*	*Comparison*	ΔG^2	Δdf	*p*
(7)	(language × phrasing × order)	0	0	1					
(6)	(language × phrasing) & (language × order) & (phrasing × order)	4.015	4	.404	language × phrasing × order	(6) – (7)	4.015	4	.404
(5)	(language × phrasing) & (language × order)	4.112	5	.533	phrasing × order	(5) – (6)	0.097	1	.755
(4)	(language × phrasing) & order	10.660	9	.300	language × order	(4) – (5)	6.548	4	.162
(3)	(language × phrasing)	11.956	10	.288	order	(3) – (4)	1.296	1	.255
(2)	language & phrasing	17.484	14	.231	language × phrasing	(2) – (3)	5.528	4	.237
(1)	language	28.418	15	.019	phrasing	(1) – (2)	10.934	1	<.001
(0)	*null-logit*	73.242	19	<.001	language	(0) – (1)	44.824	4	<.001

Supplementary Table 2.10. Log-linear analysis for the item Ass_n5 ("{*Front/Back*} of an ordered number list …).

Nr	*Model*	G^2	*df*	*p*	*Effect*	*Comparison*	ΔG^2	Δdf	*p*
(7)	(language × phrasing × order)	0	0	1					
(6)	(language × phrasing) & (language × order) & (phrasing × order)	15.393	12	.221	language × phrasing × order	(6) – (7)	15.393	12	.221
(5)	(language × phrasing) & (language × order)	19.262	15	.202	phrasing × order	(5) – (6)	3.869	3	.276
(4)	(language × phrasing) & order	33.593	27	.178	language × order	(4) – (5)	14.331	12	.280
(3)	(language × phrasing)	38.789	30	.131	order	(3) – (4)	5.196	3	.158
(2)	language & phrasing	60.521	42	.032	language × phrasing	(2) – (3)	21.732	12	.041
(1)	language	93.353	45	<.001	phrasing	(1) – (2)	32.832	3	<.001
(0)	*null-logit*	202.353	57	<.001	language	(0) – (1)	109.000	12	<.001

Supplementary Table 2.11. Log-linear analysis for the item Ass_α4 ("{*Front/Back*} of the English alphabet …).

Nr	*Model*	G^2	*df*	*p*	*Effect*	*Comparison*	ΔG^2	Δdf	*p*
(7)	(language × phrasing × order)	0	0	1					
(6)	(language × phrasing) & (language × order) & (phrasing × order)	11.268	12	.506	language × phrasing × order	(6) – (7)	11.268	12	.506
(5)	(language × phrasing) & (language × order)	13.622	15	.554	phrasing × order	(5) – (6)	2.354	3	.502
(4)	(language × phrasing) & order	26.988	27	.464	language × order	(4) – (5)	13.366	12	.343
(3)	(language × phrasing)	31.122	30	.409	order	(3) – (4)	4.134	3	.247
(2)	language & phrasing	48.872	42	.216	language × phrasing	(2) – (3)	17.750	12	.123
(1)	language	66.992	45	.018	phrasing	(1) – (2)	18.120	3	<.001
(0)	*null-logit*	177.194	57	<.001	language	(0) – (1)	110.202	12	<.001

Supplementary Table 2.12. Log-linear analysis for the item Ass_w ("{*Front/Back*} of the word "holiday" …).

Nr	*Model*	G^2	*df*	*p*	*Effect*	*Comparison*	ΔG^2	Δdf	*p*
(7)	(language × phrasing × order)	0	0	1					
(6)	(language × phrasing) & (language × order) & (phrasing × order)	11.727	12	.468	language × phrasing × order	(6) – (7)	11.727	12	.468
(5)	(language × phrasing) & (language × order)	11.971	15	.681	phrasing × order	(5) – (6)	0.244	3	.970
(4)	(language × phrasing) & order	32.427	27	.217	language × order	(4) – (5)	20.456	12	.059
(3)	(language × phrasing)	33.586	30	.298	order	(3) – (4)	1.159	3	.763
(2)	language & phrasing	78.435	42	.001	language × phrasing	(2) – (3)	44.849	12	<.001
(1)	language	93.906	45	<.001	phrasing	(1) – (2)	15.471	3	.001
(0)	*null-logit*	220.038	57	<.001	language	(0) – (1)	126.132	12	<.001

Supplementary Table 2.13. Log-linear analysis for the item Ass_q ("{*Front/Back*} of a questionnaire …).

Nr	*Model*	G^2	*df*	*p*	*Effect*	*Comparison*	ΔG^2	Δdf	*p*
(7)	(language × phrasing × order)	0	0	1					
(6)	(language × phrasing) & (language × order) & (phrasing × order)	7.770	12	.803	language × phrasing × order	(6) – (7)	7.770	12	.803
(5)	(language × phrasing) & (language × order)	10.018	15	.819	phrasing × order	(5) – (6)	2.248	3	.523
(4)	(language × phrasing) & order	29.905	27	.318	language × order	(4) – (5)	19.887	12	.069
(3)	(language × phrasing)	30.735	30	.429	order	(3) – (4)	0.830	3	.842
(2)	language & phrasing	50.240	42	.179	language × phrasing	(2) – (3)	19.505	12	.077
(1)	language	55.305	45	.140	phrasing	(1) – (2)	5.065	3	.167
(0)	*null-logit*	129.151	57	<.001	language	(0) – (1)	73.846	12	<.001
